# Mammalian Annotation Database for improved annotation and functional classification of Omics datasets from less well-annotated organisms

**DOI:** 10.1093/database/baz086

**Published:** 2019-07-26

**Authors:** Jochen T Bick, Shuqin Zeng, Mark D Robinson, Susanne E Ulbrich, Stefan Bauersachs

**Affiliations:** 1 Animal Physiology, Institute of Agricultural Sciences, ETH Zurich, Zurich, Switzerland; 2 Genetics and Functional Genomics, Vetsuisse Faculty Zurich, University of Zurich, Zurich, Switzerland; 3 Institute of Molecular Life Sciences and SIB Swiss Institute of Bioinformatics, University of Zurich, Zurich, Switzerland

## Abstract

Next-generation sequencing technologies and the availability of an increasing number of mammalian and other genomes allow gene expression studies, particularly RNA sequencing, in many non-model organisms. However, incomplete genome annotation and assignments of genes to functional annotation databases can lead to a substantial loss of information in downstream data analysis. To overcome this, we developed Mammalian Annotation Database tool (MAdb, https://madb.ethz.ch) to conveniently provide homologous gene information for selected mammalian species. The assignment between species is performed in three steps: (i) matching official gene symbols, (ii) using ortholog information contained in Ensembl Compara and (iii) pairwise BLAST comparisons of all transcripts. In addition, we developed a new tool (AnnOverlappeR) for the reliable assignment of the National Center for Biotechnology Information (NCBI) and Ensembl gene IDs. The gene lists translated to gene IDs of well-annotated species such as a human can be used for improved functional annotation with relevant tools based on Gene Ontology and molecular pathway information. We tested the MAdb on a published RNA-seq data set for the pig and showed clearly improved overrepresentation analysis results based on the assigned human homologous gene identifiers. Using the MAdb revealed a similar list of human homologous genes and functional annotation results regardless of whether starting with gene IDs from NCBI or Ensembl. The MAdb database is accessible via a web interface and a Galaxy application.

## Introduction

In transcriptomics and proteomics studies, one important step of data analysis is the functional annotation of obtained lists of differentially expressed genes (DEGs) or proteins (DEPs). With the increase in the number of organisms with sequenced and annotated genomes, such studies have been performed in many different species. However, the information about gene and protein functions, on which the annotation is based, is mainly derived from a limited number of model organisms or well-annotated species, such as mouse, humans and rat representing mammalian species or even from bacteria, yeast, worms or Drosophila. Based on the assumption that orthologous genes carry out identical or biologically equivalent functions in different organisms, functional annotation is transferred from well-studied organisms to less well-annotated species (1). Whereas orthologous genes usually have maintained the same function during evolution (2) paralogous genes originated from gene duplication events and often evolved different functions (3, 4). Orthologous genes usually have the same gene symbol and name for almost all corresponding species (5, 6). However, depending on the status of the gene annotation of a species, not all annotated genes have an official gene symbol (only locus number, e.g. LOC100152218 60S ribosomal protein L23a-like) and/or are assigned to functional annotation databases like their corresponding orthologs in the well-annotated model organisms. This leads to a substantial loss of information if the gene identifiers (IDs) of the respective species are used for functional annotation. To avoid this data loss and improve the results of functional annotation, one strategy is to transfer information from homologous genes (orthologs and paralogs) of well-annotated species (6).

In many situations with non-model species such as livestock species, experimentalists avoid the additional work to assign human ortholog genes for functional annotation analysis and are not aware of the information loss. For example, of the first 10 hits of a search in PubMed with ‘*Sus scrofa* AND RNA-seq’ (7–16), seven performed typical Gene Ontology and/or pathway enrichment analysis (17,18). One of those studies used the STRING database (19) with porcine Ensembl IDs and Blast2GO (20), the latter an approach similar to the Basic Local Alignment Search Tool (BLAST) (21) step of our tool. Another study stated in the Materials and Methods the conversion to human gene IDs without any details of how it was performed. The remaining five out of seven studies used porcine gene IDs for functional annotation and did not convert to human IDs. Also for other species, only a few studies have considered this issue, e.g. a study in salmon using a homology approach for functional annotation and pathway analysis (22).

A number of existing databases contain information regarding orthologous genes, e.g. Ensembl Compara ortholog database (Ecodb) or OMABrowser (23, 24). These databases are constructed in three distinct ways: tree based, graph based or using meta-methods (25). More importantly, they are based on different source databases such as Ensembl, UniProt, EBI or National Center for Biotechnology Information (NCBI) (see Table 1). Furthermore, one important issue is the update cycle and version of such databases, because it is essential to use the most up-to-date information corresponding to genome assemblies and annotation for transcriptome or proteome studies. For some databases, the update cycles are not very frequent and/or regular because they are related to a running project with timely limited funding (see Table 1). Therefore, depending on the type of analysis, existing databases have their limitations.

**Table 1:** Overview of ortholog databases available for public usage (data collected 23 January 2019)

Ortholog database	Comment	Main source	Method	Version	Last update	Webpage
Ensembl Compara	15 270 Genomes	Ensembl	Tree based	Release 95	2019–01	http://www.ensembl.org
PANTHER	131 Genomes	UniProt ID mapping	Tree based	PANTHER14.0	2018–04	http://www.pantherdb.org
PhylomeDB	all Genomes	Ensembl, UniProt	Tree based	Version 4	2014–01	http://phylomedb.org
Best Reciprocal Hits	Only bacteria	NCBI	Graph based	–	–	–
Reciprocal Smallest Distance	–	–	Graph based	–	–	–
EggNOG	5090 Organisms	na	Graph based	Version 5.0	2018–11	http://eggnogdb.embl.de
Hieranoid	66 Species	EBI, UniProt	Graph based	Version 2	2016–04	http://hieranoid.sbc.su.se
InParanoid	273 Organisms	UniProt	Graph based	Version 8.0	2013–12	http://inparanoid.sbc.su.se
OMABrowser	2103 Species	Ensembl, UniProt	Graph based	na	2018–12	https://omabrowser.org/
OrthoInspector	4753 Species	UniProt	Meta-method	v3	2018–10	https://lbgi.fr/orthoinspectorv3/
MetaPhOrs	2714 Species	Ensembl	Tree- and graph based	v.2	2018–04	http://betaorthology.phylomedb.org/
DIOPT	10 Organisms	Multiple sources (18)	–	Version 7.1	2018–03	https://www.flyrnai.org/cgi-bin/DRSC_orthologs.pl/
Isobase	5 Species	–	–	v.2	2014–11	http://cb.csail.mit.edu/cb/mna/isobase/
Treefam	109 Species	–	Tree based	Release 9	2013–03	http://www.treefam.org/
Wormhole	6 Species	Ensembl	–	–	2016–11	http://wormhole.jax.org/
GreenPhyl	37 Plants	–	–	Version 4 update 1	2015–09	http://www.greenphyl.org/cgi-bin/index.cgi
bioDBnet	All genomes	NCBI, Ensembl, UniProt	Meta-method	–	2019–01	https://biodbnet-abcc.ncifcrf.gov/

To overcome the limitation of individual annotation databases, a convenient solution could be to combine the information obtained from different databases. However, one major issue with this approach is to correctly assign different gene, transcript or protein IDs, which are based on the same genome assembly information but derive from different annotation platforms such as Ensembl or NCBI. For example, the Ecodb does not directly provide NCBI Entrez Gene IDs and the assignment available in Ensembl BioMart (26, 27) is incomplete and contains errors (28, 29). However, since many researchers are working with the resources provided by the NCBI and many available tools for functional annotation do not work with Ensembl gene IDs, information from other databases has to be reliably converted to Entrez Gene IDs. In order to provide a comprehensive and reliable tool for assigning orthologous gene information, we developed the Mammalian Annotation Database (MAdb). This database tool is based on three steps to assign orthologs or paralogs or at least genes with substantial sequence similarity: (i) comparing official gene symbols, (ii) retrieving and filtering orthologous gene information from Ensembl Compara and (iii) integrating pairwise BLAST comparisons of all annotated transcripts of the included species. Steps 2 and 3 can also deliver other homologous genes such as paralogs. However, to simplify we were using in the following just the term ‘orthologs.’ The assignment of the corresponding Entrez Gene IDs to Ensembl gene IDs turned out to be a particular problem. For example, existing mappings that assign corresponding gene pairs, such as gene2ensembl provided by NCBI (30, 31), Ensembl BioMart (27) or UniProt (32, 33), are incomplete and/or contain errors.

The hypothesis that using orthologous gene IDs (and paralogous gene IDs if no ortholog is present) of classical model organisms improves the functional annotation results over the corresponding species own IDs was tested using an RNA-seq data set derived from an analysis of porcine endometrium (34). Furthermore, the MAdb was compared to existing databases for orthologous gene information. To achieve a genome position-based assignment of the two annotation sources (NCBI and Ensembl), a combination of R BioConductor packages (35) was used to analyze overlapping gene and exon positions that were integrated as a lookup table into a MySQL database. Analyzing a sample data set, we could highlight the benefits of using our database. Moreover, the MAdb tool provides a basis for cross-species comparisons of transcriptome data sets from different mammalian species. The MAdb database is available online (36) (https://moadb.ethz.ch) and integrated as an app (37) that will be soon available in the ToolShed (38) of Galaxy to give easy access to other research groups.

## Materials and methods

### Gene symbol match across species

The gene_info file from NCBI (39) (download date: 10 January 2019) was used as an information source for the ortholog assignment based on identical gene symbols. This was achieved by filtering the gene_info file on official HUGO Gene Nomenclature Committee (HGNC) gene symbols (40, 41), of the seven mammalian species included in the initial version of the database and finally collapsing on gene symbols (download date: 10 January 2019). Additionally, a quantitative trait locus (QTL) filter was introduced to remove QTLs, which have, in some cases, symbols identical to unrelated HGNC gene symbols.

### Retrieving data sets from NCBI and Ensembl to perform a positional overlap

In the first step of the assignment of annotated genes between NCBI and Ensembl, the genome annotation files of seven mammalian species (human, mouse, cow, pig, horse, dog and rabbit) were downloaded from two different databases (NCBI and Ensembl) (download date: 10 January 2019). The NCBI stores their annotation information preferentially in generic feature format (GFF) (42) files while Ensembl uses the general transfer file/format (GTF) (43). These two file types share basically the same common information including features of genes, exons and coding sequences (CDS) in a tab-delimited table format whereby the main differences are in the column ‘attributes’ (44). The automated pipeline downloads and harmonizes the two annotation files for the same genome assembly (Table 3, column ‘Assembly’). For some species, the assembly versions are different between NCBI and Ensembl due to different update cycles. For the annotation overlapper approach AnnOverlappeR (AOR) (see below), the assembly versions have to be identical.

**Figure 1:**
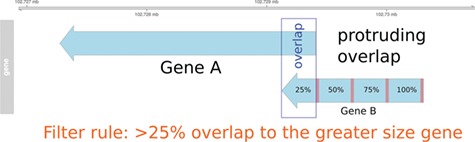
Pre-filtering of overlapping genes. In this example, two genes (A and B) annotated at the same genomic position at NCBI and Ensembl are compared to match the identifiers. In the case of a protruding overlap, the cut-off is set to 25% for the smaller sized overlapping gene (gene B). Overlaps <25% are dismissed by the overlapping approach.

### Approach for comparison of NCBI and Ensembl genome annotation

In order to use information from the Ensembl Compara database and to enable the use of the MAdb for datasets with Ensembl gene IDs, a position-based approach was developed [AnnOverlappeR, available at GitHub (37)] to assign corresponding genes between the NCBI and the Ensembl gene annotation for the same genome assembly. The AOR is based on a modification of the method used by NCBI to generate the gene2ensembl file to assign the corresponding gene annotated by the Ensembl annotation pipeline. The NCBI method checks if annotated RNA and CDS features overlap at least 80%. Furthermore, NCBI checks whether at least 60% of the splice sites are matching, or if there is at most one splice site mismatch (45). In contrast, the AnnOverlappeR uses less conservative parameters and a filtering consisting of three steps to increase the number of assigned genes but at the same time avoiding false positives (37), (i) analysis of overlaps at the gene level, (ii) overlap of exon and CDS positions and (iii) filtering and validation of overlaps according to a certain cut-off (>50% overlap). All steps were performed using Bioconductor R packages (46). Annotation information was imported with the package GenomicRanges (47). These ranges were filtered and split into different features of interest: genes, exons and CDS. Since GFF and GTF files contain different additional information in the attributes, a processing step was necessary to make them comparable. In the following step, an overlap function was run at gene level using GenomicAlignments::findOverlaps(). In the first filtering step, genes that were overlapping not only within the boundaries of the larger gene, which means that genes are partially or protruding overlapping each other (Figure 1), had to overlap at least 25% with respect to the smaller sized gene. Genes with an overlap >50% were directly considered as positive hits. All remaining overlapping genes were used for the second overlapping step at the exon and CDS level. Therefore, the average exon and CDS overlap was calculated. This was done by calculating and using the inner and outer width of each overlapping element using pintersect() and punion(), two functions of the package GenomicAlignments (see source code on github). Only overlapping features with a minimum gene overlap of one base were used for the mean overlap. In the final filtering step, all overlapping genes were filtered according to a cut-off on exon and CDS level. These genes had to have an exon or CDS overlap >50%. The resulting identifier list was then filtered to remove duplicates and saved as a lookup table for the MAdb. The duplication filter finds IDs that were mapped to more than one gene ID from the respective other annotation pipelines. This was necessary to ensure that genes located in the same genomic range were assigned to the correct corresponding gene of the other annotation source (Additional file 1: Fig. S1 and S2). All filters were tested on examples, evaluated and validated for the correctness of the resulting ID pairs.

### Download and filtering of Ensembl Compara data

Every 3 months, Ensembl releases an update of the entire database. In this study, the release 95 (date of release: 7 January 2019) with the following sub-tables was used: gene_member, homology, homology_member and genome_db. These files are available on the Ensembl FTP server (48). All files were downloaded and filtered for the selected seven mammalian species. For easy usage, the ortholog table named ‘all_ortholog**’** was calculated and structured as follows: species name, stable id, entrez gene id, gene symbol, percent coverage, percent identity, percent position hit, ortholog description, chromosome, gene description, type of gene and database source. This table contained all orthologous Ensembl IDs and their assigned corresponding NCBI Entrez Gene IDs. The tables were indexed to ensure quick access.

### BLASTn comparison of transcriptomes

Nucleotide BLAST was used in order to assign also non-coding genes. The latest current RefSeq transcript fasta files were downloaded from NCBI (49) (download date: 10 January 2019). All seven species so far included in the MAdb were aligned against each other using BLASTn (21) with several specific options (Additional file 1: Table S1) to identify the best matching transcript of the compared species. The resulting BLAST output files were then filtered for a minimal bit score, query coverage and best hit (based on bit score). While for the current MAdb version transcript sequences (i.e. for many genes more than one sequence) were compared, joined gene exon sequences will be used in future database updates.

### Online access to the database

The MAdb was implemented in an online search tool to give access to the research community. Users have two options to obtain information. The user can upload a list of selected genes in three formats: Entrez Gene IDs, Ensembl gene IDs or HGNC gene symbols. Alternatively, a species can be selected to include all genes of this particular species. To retrieve ortholog information of other species, one or more mammalian species can be selected. In the final step, the output fields of the resulting table can be selected.

### Integration of the MAdb as a Galaxy tool

The implementation of the Galaxy wrapper strictly follows the online access to the MAdb from a Galaxy server. The Galaxy tool was designed in such a way that it is able to handle chosen gene lists generated within Galaxy to retrieve ortholog information without exporting from Galaxy. The tool itself is redirecting to the webpage of the MAdb and collecting the selected data that was parsed back to the Galaxy server. Uploaded gene lists must be in tabular or text format and can be transferred to the MAdb web page for further processing. The output format of this tool is also a tabular format that can be opened in Microsoft Excel. Users should always be careful when opening the text file in Excel to set the column format for gene symbols always to ‘text.’ Otherwise, a number of symbols (e.g. SEPT1, septin 1) will be automatically converted by Excel to dates (50).

### Update cycle MAdb

The MAdb will be frequently updated. Since two parts of the MAdb pipeline are depending on the Ensembl database (Ensembl Compara and the GTF file for the AOL), it is reasonable to update the MAdb at the same time as new Ensembl releases appear, i.e. every 3 months. In addition, it is necessary to check if the genome assembly versions of the currently available species are identical for NCBI and Ensembl.

**Figure 2:**
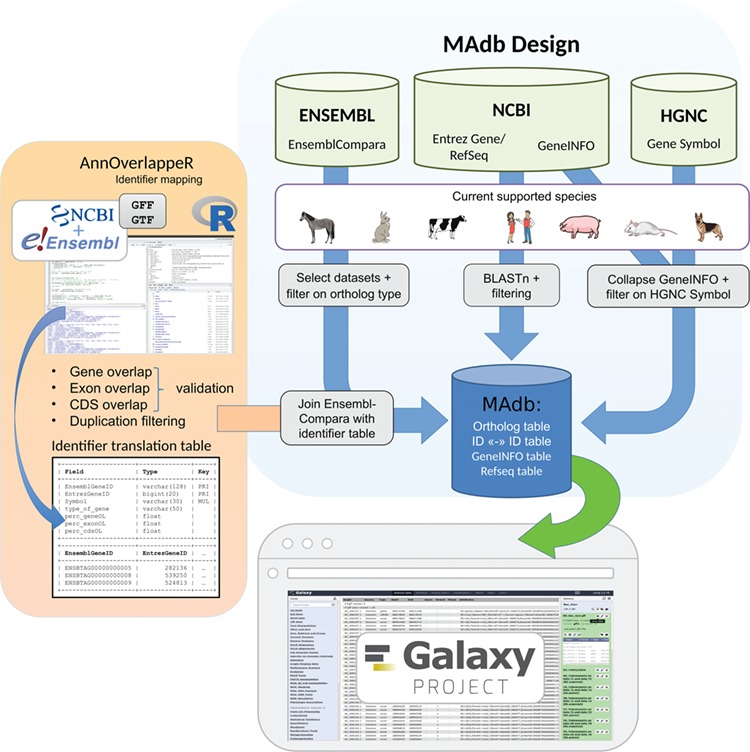
Schema of the MAdb pipeline. The pipeline is split into three parts. In the part highlighted in blue, the data collection from three different databases (Ensembl, NCBI and HGNC) is shown. Selected, filtered data sets of these three databases are the basis of the MAdb. Each data source is filtered on the currently supported species. Additionally, Ensembl Compara is filtered on selected database subtables and ortholog types within the data tables. The NCBI provides Entrez Gene and RefSeq information to do pairwise BLAST comparisons and the GeneINFO file used for gene symbol matching. The results are then filtered for the official gene symbols from HGNC. In orange, the identifier overlapping approach (AnnOverlappeR) is presented. First, GFF and GTF annotation files are collected from NCBI and Ensembl. The overlapping approach is based on gene, exon and CDS overlaps and a final duplication filter. A lookup table is generated that is used to connect Ensembl data sets with NCBI data sets. The MAdb is accessible as a Galaxy app and an online tool at https://madb.ethz.ch/ (highlighted in gray).

### Mapping of the RNA-seq example data set and detection of DEGs

An example Illumina RNA-seq data set for the pig was used (NCBI GEO: GSE43667). This data set consisted of eight samples from porcine (*Sus scrofa*) endometrium (Day 14 of pregnancy, *n* = 4, and Day 14 of the estrous cycle, *n* = 4). The processing and mapping of Illumina short reads was performed on a local Galaxy installation (51). A standard RNA-seq workflow was used including trimming, quality control and mapping (HiSAT2) (52) to the latest porcine genome assembly (Sscrofa11.1) and QuasR qCount (53) for all annotated porcine genes (NCBI GFF3 file: (54) and Ensembl GTF file: (55)). Afterward, filtered read counts were used to identify DEGs in edgeR (56). Filtering was performed based on a CPM cut-off, i.e. genes passing the filter had to have 1.66 CPM or more (20 or more reads) in at least three libraries, to be retained for differential expression analysis. The read count data were normalized to the library size and trended dispersions. Finally, the statistical analysis was performed for pregnant versus control. DEGs were filtered on a false discovery rate (FDR) of 1%.

**Table 2:** Gene symbol matches per species: number of genes with official gene symbol (HGNC symbols) and percentage of matches of genes with official gene symbol between species

Species	Number of genes (NCBI)^a^	Number of HGNC gene symbols	Bta	Cfa	Eca	Hsa	Mmu	Ocu	Ssc
Bta	37 862	17 507	100	93.61	93.49	99.33	90.72	84.79	92.69
Cfa	37 701	16 761	97.77	100	96.25	99.78	92.71	87.66	95.37
Eca	33 571	16 740	97.77	96.37	100	99.69	92.57	87.89	95.66
Hsa	60 283	40 803	42.62	40.99	40.90	100	40.96	36.91	40.34
Mmu	68 711	16 728	94.94	92.89	92.64	99.92	100	84.79	92.06
Ocu	30 874	15 106	98.27	97.27	97.39	99.71	93.89	100	97.25
Ssc	31 325	16 545	98.08	96.62	96.78	99.47	93.08	88.79	100

a ^a^Based on the gene_info file from NCBI after removing entries with the type of gene ‘unknown.’ Species are presented in three letter code, e.g. *Bos taurus* = Bta.

### Functional annotation analysis

A functional annotation analysis was performed using the ‘Functional Annotation Clustering’ and ‘Functional Annotation Charts’ tool of the Database for Annotation, Visualization and Integrated Discovery (DAVID, version 6.8) (57). The functional annotation analysis was performed separately for up- and downregulated genes starting the RNA-seq data analysis from the NCBI GFF3 file (with Entrez Gene IDs) and from the GTF file from Ensembl (with Ensembl gene IDs), respectively. For the comparison to other ortholog databases, four different strategies to retrieve ortholog gene information were used: (i) Ensembl BioMart plus Ensembl Compara (23), (ii) OMABrowser (with given IDs), (iii) OMABrowser plus AnnOverlappeR (translated Entrez Gene IDs) and (iv) using the MAdb tool. In addition, porcine Entrez Gene IDs were used for DAVID. The corresponding gene ID lists were uploaded to DAVID using RDAVIDWebService (58). As annotation categories, GOTERM_BP_FAT, GOTERM_CC_FAT and GOTERM_MF_FAT were selected from Gene Ontology and KEGG_PATHWAYS for pathways (Functional Annotation Clustering modified parameters: similarity threshold = 0.6, enrichment thresholds 0.2 and for the Functional Annotation Charts the parameters were set as follows: Count = 1, EASE = 1).

## Results

### Structure of the MAdb

The basic idea of the database for the assignment of orthologous genes was to use information derived from existing databases and to complement this information with the results of pairwise BLAST comparisons of the individual transcriptomes for the species contained in the database (see schematic overview in Figure 2). In the first step of assigning orthologs, HGNC gene symbols were compared between species to assign the corresponding Entrez Gene IDs. Since for many species a considerable number of genes still have only locus numbers (e.g. LOC100127131) as provisional gene symbols or gene symbols for some genes are not the same for all species despite they are orthologous genes, additional data sources have to be used. The Ensembl Compara database and results from BLAST comparisons of the transcriptomes were used to increase the number of assigned orthologs (including known and potential orthologs).

With respect to the database structure, the MAdb consists of a number of sub-tables. The main table is based on the three different data sources as mentioned above. Table 2 shows the numbers of genes in the NCBI GENE_INFO tables after removing entries that actually do not represent genes (QTLs, promoter/enhancer regions, deletions, translocations, minisatellites etc.), the numbers of HGNC symbols and the numbers of symbols matching to each other species in percent.

**Table 3:** Content of NCBI (GFF) and Ensembl (GTF) annotation files and percentage of assigned genes between Entrez Gene and Ensembl

Species	Assembly	NCBI genes (GFF)	Ensembl genes (GTF)	Overlaps	Validated	IDs from NCBI	IDs from Ensembl	% NCBI	% Ensembl
Bta	ARS-UCD1.2	35 158	27 570	29 301	24 208	23 470	23 749	66.8	86.1
Cfa	CanFam3.1	36 945	32 704	29 094	23 144	22 273	22 624	60.3	69.2
Eca	EquCab3.0	33 146	31 217	28 377	23 758	22 969	23 046	69.3	73.8
Hsa	GRCh38.p12	60 959	58 735	68 598	47 307	42 869	42 899	70.3	73.0
Mmu	GRCm38.p6	48 737	54 838	49 655	37 193	35 090	35 078	72.0	64.0
Ocu	OryCun2.0	29 480	23 669	22 748	19 715	19 427	19 231	65.9	81.2
Ssc	Sscrofa11.1	30 364	25 880	26 729	22 168	21 206	21 038	69.8	81.3
Total/average					197 493			67.8	75.5

**Figure 3:**
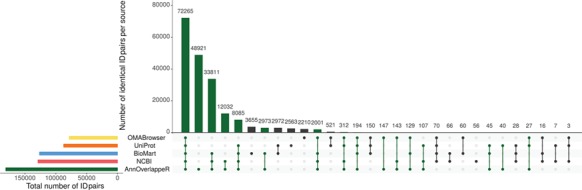
Overlap of identifier matches between NCBI and Ensembl for different identifier mappers. This Upset plot (73) shows the overlap of five different identifier mappers (AnnOverlappeR, Biomart, NCBI, OMABrowser and Uniprot) for all MAdb species. Highlighted in green: ID pairs detected with the AnnOverlappeR.

**Table 4:** NCBI genes in GFF annotation file assigned to Ensembl gene IDs: Comparison of the percentage of assigned Entrez Gene IDs present in the GFF file for each ID mapper

Species	AOR	NCBI	BIOMART	UNIPROT	OMA
Bta	66.8	56.3	45.3	27.8	27.2
Cfa	60.3	43.6	46.1	26.6	26.1
Eca	69.3	54.1	45.5	14.9	16.5
Hsa	70.3	42.4	42.1	31.3	29.0
Mmu	72.0	50.8	43.8	42.7	41.7
Ocu	65.9	47.7	47.3	26.0	25.2
Ssc	69.8	57.2	61.9	24.2	43.4

The basis for the extraction of ortholog data from Ensembl Compara is a lookup table ‘ensembl2entrezgene’ containing the result of the tool AOR. This tool links Entrez Gene and Ensembl gene IDs based on genomic positions and overlap of annotated exons. Using the ‘ensembl2entrezgene’ table, the Ensembl Compara ortholog information is assigned to the corresponding Entrez Gene IDs. As shown in Table 3, NCBI and Ensembl contain different numbers of annotated genes for the same genome assembly because of using different annotation pipelines. For the seven selected species (except for the mouse) the NCBI annotation contained a higher number of annotated genes. On average, ~68% of all NCBI gene IDs were assigned to Ensembl and 75% of the Ensembl IDs to NCBI (Table 3). In comparison to the other sources, AOR detected within total 191 495 ID pairs the by far highest number of ID pairs, followed by NCBI (gene2ensembl, 135 967 ID pairs), Ensembl BioMart (132 940 ID pairs), OMABrowser (83 849 ID pairs) and Uniprot (82 632 ID pairs) (Figure 3). The AOR found 49 559 unique ID pairs that were not detected by any other method. This is additionally shown in Table 4 with respect to the assigned Entrez Gene IDs. A comparison of gene overlap versus exon or CDS overlap showed that most of the novel ID pairs had a gene overlap of >90% (Additional file 1: Figure S3; red hexagon in the right corner). Additional novel ID pairs had either a high exon or CDS overlap or even a high gene overlap too (see cyan hexagons upper left and right corner of Additional file 1: Figure S3). Furthermore, a number of cases are shown where it is not simple to find the correct corresponding gene in an automated way (Additional file 1: Figures S4–S12). The ID pairs not found with AOR but by the other sources were assessed for correctness (Additional file 1: Figure S13). Gene2ensembl (NCBI) contained with 56 the lowest number of ID pairs not contained in any other database. Much higher numbers of unique ID pairs were found in Ensembl BioMart, UniProt and OMABrowser (Figure 3). For almost half of the ID pairs (approximately 5000 ID pairs) not found by AOR, the genomic location of the corresponding Entrez Gene and Ensembl genes was not overlapping. That means they were either located at a completely different location on the same chromosome or even on different chromosomes. Thus, this large number of ID pairs clearly represents false positives. Other ID pairs not found with AOR were simply not included in the NCBI GFF file (approximately 250 ID pairs) or in the Ensembl GTF file (approximately 5000 ID pairs) or even not present in both files (approximately 10 ID pairs). A neglectable proportion of ID pairs (approximately 250) was lost due to the filter for duplicates or removed because the overlap between the NCBI and Ensembl annotation (approximately 100 ID pairs) was too small.

Furthermore, BLASTn comparisons of the transcriptomes between the species of the MAdb were the third source for homologous genes in addition to the assignment by gene symbol match and Ensembl Compara information.

**Figure 4:**
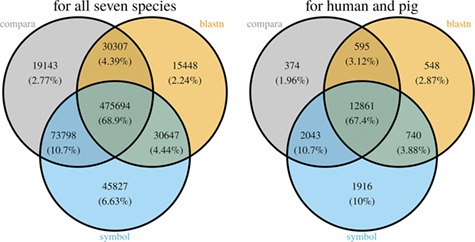
Comparison of the number of genes assigned to the three MAdb data sources. The left Venn diagram shows the sum of assigned genes between all seven species for the three MAdb data sources. The second (right) Venn diagram shows the number of genes assigned between human and pig for all the three MAdb data sources.

With the species currently included in MAdb, ~89% of all protein-coding genes (according to NCBI’s Gene_info) exhibit an ortholog match to at least one other species (Additional file 1: Table S2). Matches between all seven species in the MAdb were obtained for ~69% of the protein-coding genes. Part of the known or putative (BLASTn approach) orthologous gene relationships was also uniquely detected by one of the three approaches (~7% for the gene symbol matcher, ~2% for BLASTn and ~3% for Ensembl Compara, see Figure 4). Similar results were obtained for genes assigned for human and pig (see Figure 4). For other gene types, such as non-coding genes, the number of orthologs was smaller (Additional file 1: Table S2). The total number of orthologous genes accessible by MAdb is shown in Table 5.

**Table 5:** Homologous genes (including also paralogs and orthologs) represented by MAdb: number of matched genes between each species represented by the MAdb

Species	Bta	Cfa	Eca	Hsa	Mmu	Ocu	Ssc
Bta	0	23 998	25 085	25 729	27 128	24 936	27 340
Cfa	23 400	0	23 533	24 946	23 303	23 027	24 698
Eca	24 574	23 550	0	24 156	24 935	23 697	25 699
Hsa	26 474	26 137	25 714	0	28 407	25 614	27 654
Mmu	27 382	24 575	25 590	28 844	0	27 725	29 726
Ocu	24 157	23 168	23 368	24 649	26 669	0	26 435
Ssc	26 917	24 763	25 616	26 305	28 479	26 449	0

### Comparison of DAVID Functional Annotation results using original species’ gene IDs versus the IDs of human orthologs

**Figure 5:**
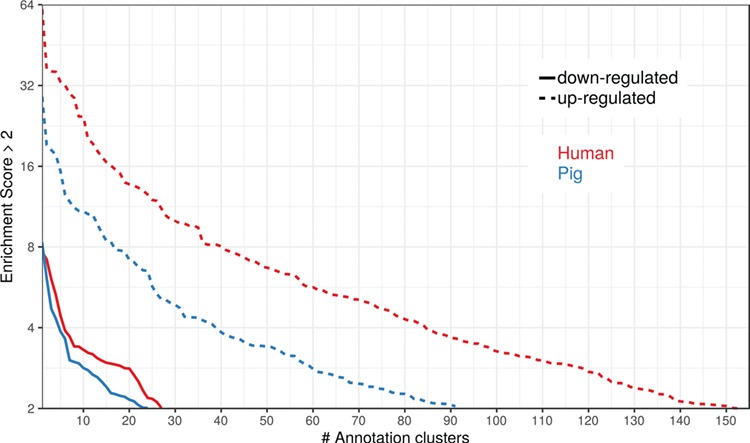
Number of DAVID functional annotation clusters plotted against the enrichment score for assigned human gene IDs and the original porcine gene IDs. A cut-off of 2 for the enrichment score was used (geometric mean of the *P* value of the categories in an annotation cluster ≤0.01). Results are shown for upregulated (dashed line) and downregulated (solid line) genes.

**Figure 6:**
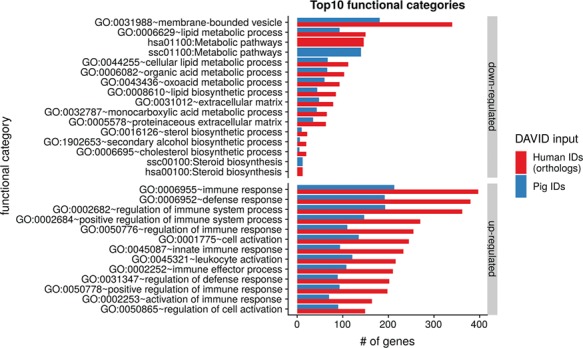
Top 10 DAVID functional annotation categories for human and porcine gene IDs. The numbers of assigned genes to the top 10 functional categories obtained from DAVID GO chart analysis (top 10 of each database collected) are shown for up- and downregulated genes, respectively.

**Figure 7:**
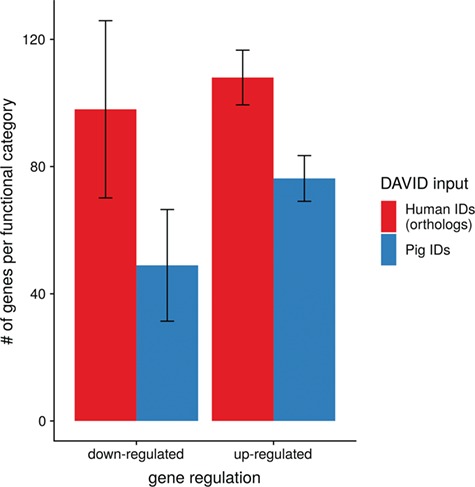
Average number of genes for functional annotation categories obtained for up- and downregulated genes for human and pig gene IDs.

To show the benefit of using human orthologous gene IDs for functional annotation of DEG lists, an RNA-seq dataset (porcine endometrium samples) published by Samborski *et al*. (2013) was reanalyzed based on the current porcine genome assembly and annotation (Sscrofa11.1). In total, 3132 genes were found as differentially expressed at an FDR of <1%, 1805 with higher expression (upregulated) and 1327 with lower expression (downregulated) in comparison of samples from Day 14 of pregnancy and samples from Day 14 of the estrous cycle (nonpregnant control). MAdb retrieved 3068 human gene IDs. DAVID Functional Annotation Clustering was performed for the DEGs using the porcine Entrez Gene IDs and the human Entrez Gene IDs, respectively, and the obtained results were compared. DAVID Functional Annotation Clustering resulted for the human gene ID list in 153 and 27 annotation clusters (for up- and downregulated genes, respectively) with an enrichment score of >2. In contrast, the DAVID analysis starting from the porcine Entrez Gene IDs revealed only 91 and 24 annotation clusters (Figure 5). Furthermore, the highest enrichment score for upregulated genes was ~62 for human IDs compared to approximately 29 for porcine IDs. DAVID Functional Annotation Chart analysis was also performed for the up- and downregulated DEGs with porcine and human identifiers, respectively. The 10 most significant functional categories were compared. The number of associated genes per category was much higher for human IDs (Figure 6). In general, we could show that the average number of genes per annotation cluster/functional category was significantly higher using human gene IDs compared to porcine gene IDs (Figure 7).

### Comparison of functional annotation results using humanized porcine DEG lists between the MAdb and related databases

**Figure 8:**
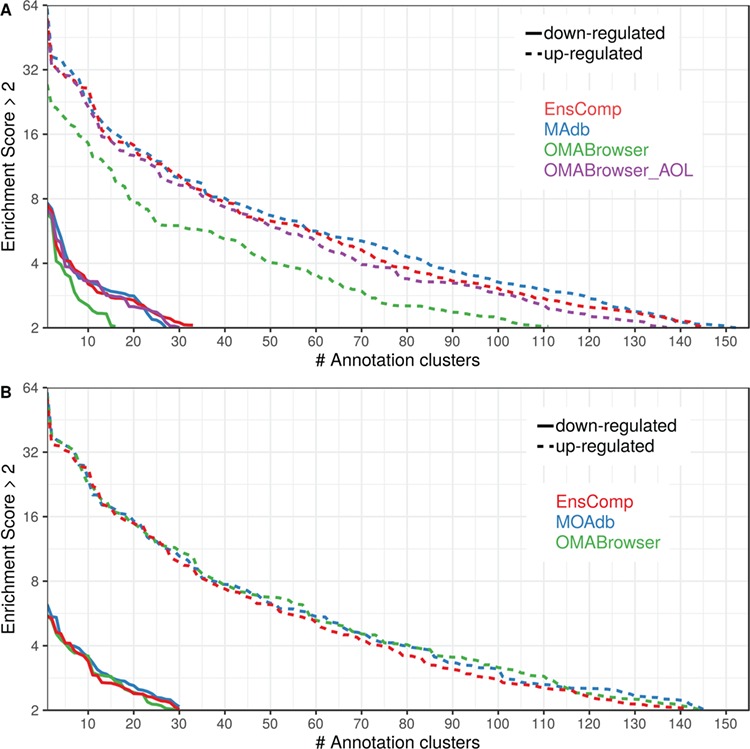
Number of DAVID functional annotation clusters plotted against the enrichment score obtained using different ortholog database sources. A shows the result by using Entrez Gene IDs, and B represents Ensembl gene IDs. A cut-off of 2 for the enrichment score was used (geometric mean of the *P* value of the categories in an annotation cluster ≤0.01). Results are shown for upregulated (dashed line) and downregulated (solid line) genes.

**Figure 9:**
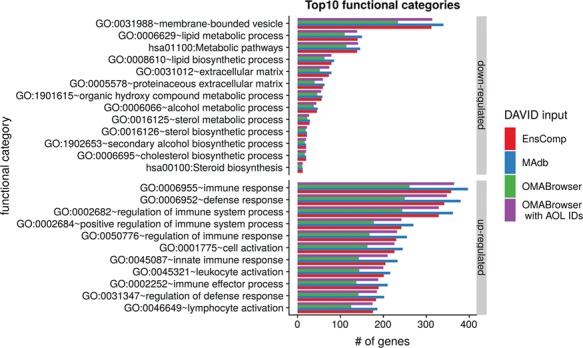
Top 10 DAVID functional annotation categories in comparison of four different databases (EnsComp: Ensembl Compara, MAdb, OMABrowser and OMABrowser with AnnOverlappeR IDs). The numbers of assigned genes to the top 10 functional categories obtained from DAVID GO chart analysis (top 10 of each database collected) for up- and downregulated genes, respectively.

The DAVID analysis based on human gene IDs derived from MAdb was compared to ortholog DEG lists obtained from (i) Ensembl BioMart plus Ensembl Compara, (ii) OMABrowser and (iii) OMABrowser plus AOR. The numbers of assigned human gene IDs are shown in Additional file 1: Figure S14. The highest number of exclusively detected human gene IDs (HOG) was obtained from MAdb (103 HOG), followed by the combination of OMABrowser and AnnOverlappeR (36 HOG), Ensembl Compara (28 HOG) and four HOG from OMABrowser (Figure S14A). The obtained human gene IDs were used as input for a DAVID functional annotation analysis for each database. The results of Functional Annotation Clustering with an enrichment score of >2 revealed for Ensembl Compara 145 and 33 annotation clusters (for up- and downregulated genes, respectively), OMABrowser plus AOR 137 and 30, OMABrowser 111 and 16 and the MAdb 153 and 27 annotation clusters. Starting the complete RNA-seq data analysis based on the Ensembl genome annotation (Ensembl Sscrofa11.1 GTF file), DAVID revealed 142 and 30 functional annotation clusters using Ensembl Compara (Enrichment score > 2), for OMABrowser 144 and 30 clusters and MAdb retrieved 145 and 30 clusters, respectively (Figure 8). The DAVID Functional Analysis Chart was also performed with the same databases. The number of associated genes per category was compared, and it was found that MAdb and OMABrowser with AOR had the highest number of associated genes followed by Ensembl and OMABrowser (Figure 9). Additional filtering multiple ortholog hits had to be performed to avoid artifacts in the DAVID analysis. Ensembl, as well as OMABrowser, showed some cases where one porcine gene ID was assigned to more than only one human ortholog (e.g. Entrez Gene ID: 100621538, histone H2A type 3).

## Discussion

Our new tool, MAdb, was developed to improve functional annotation of gene sets, mainly consisting of DEGs or proteins, for organisms with incomplete genome annotation based on the substitution of the original species gene ID by the ID of the corresponding human orthologous genes. Furthermore, a tool was needed to obtain orthologous gene pairs for cross-species comparisons of gene expression data sets. With respect to the functional annotation of DEGs lists, it has been shown that e.g. outdated gene function annotations have a tremendous impact on the results of pathway and gene ontology enrichment analysis (59). Likewise, incomplete assignment of genes to functional categories and molecular pathways, which is the case for many livestock and other animals leads to a significant loss of information and considerably reduces the gain of knowledge from gene and protein expression studies. The presented examples highlighted the advantages of our approach and the good performance in comparison to other tools or databases.

One central implementation of the MAdb tool is a position-based identifier matching (AOR) between NCBI gene IDs and Ensembl gene IDs to work database independently (NCBI or Ensembl) with orthologous gene information. The more sensitive and accurate assignment of gene IDs compared to other approaches prevents false positives in follow-up downstream analysis and allows the use of ortholog information derived from different databases. A similar approach to join and assign gene annotation from different sources has been published recently for the mouse genome (60). With the strategy used in our presented tool (AOR), we could find a higher number of ID pairs between NCBI and Ensembl compared to the ID pairs provided by NCBI and Ensembl, respectively. The additional ID pairs were probably derived from the more sensitive position-based approach compared to the very stringent NCBI approach. We could also show that a high number of the novel ID pairs had between 90% and 100% gene overlap but a relatively low overlap on exon or CDS because they do not have annotated exons or CDS in at least one of the annotation files (GFF or GTF). Additionally, we could increase the number of ID pairs with pairs that overlap highly on exon or CDS level. Our strategy was particularly designed to reduce false positives, i.e. AOR does not match NCBI and Ensembl IDs of genes that are located at different positions of the genome. This is often the case in other databases if the corresponding genes show high similarity. Other approaches found also ID pairs of genes that are not present in the current GTF/GFF annotation files. However, since the GFF/GTF files used for the MAdb are only important for the assignment of NCBI and Ensembl gene IDs that are needed for data extraction from Ensembl Compara, genes not annotated in a current genome assembly but present in Entrez Gene can still be assigned to corresponding orthologous genes via the other two steps of the pipeline.

Although it seems simple to match gene annotation for the same genome assembly derived from different annotation pipelines, the challenges are in the details. Depending on the species and e.g. the availability of sequenced full-length transcripts, gene annotation can differ significantly with respect to the number and size of exons between different annotation sources. For example, a gene is considered as one gene in one annotation source but at the same locus two or more genes are annotated in the other source. Furthermore, one source suggests an additional 5′ or 3′ exon separated by a large intron compared to the other source. In this case, the gene overlap between the annotation sources is relatively low but the exon and/or CDS overlap still high enough to match the genes. Since some redundant Entrez Gene IDs are present in the NCBI annotation files (rare cases, coming from ‘alternative loci’ NT_ and NW_), it is also necessary to filter the GFF files on specific chromosomes to have unique identifiers and corresponding chromosomal positions. Another problematic example is genes that share several exons, such as the protocadherin gamma cluster. To solve this problem, a duplicate filter was applied to retrieve only the best hit. A number of other difficult cases can be found in the Supplementary Data (Additional file 1: Figures S1 and S2, S4–S12).

The main application of the MAdb, the use of the corresponding human ortholog IDs as a substitute for the original species gene ID in functional annotation analysis, revealed more significantly enriched functional categories because more genes were assigned to the individual overrepresented categories. Using the original species gene IDs significantly reduces the outcome of the functional annotation analysis by missing overrepresented functional categories and pathways contained in the analyzed data set. Furthermore, using the original species’ gene IDs, not all identified DEGs that actually belong to a given functional category can be assigned. This relates to the problem of genes with provisional symbols such as e.g. LOC100127131 in species like the pig. In these cases, such genes are lost when going for deeper analysis of obtained overrepresented functional categories or pathways.

Initiatives are ongoing to generate data for functional annotation of animal genomes, such as GO-FAANG (61), whereas most of the information about gene and protein functions present in respective databases are derived from studies in the classical model organisms. The assignment of annotated genes in domestic and non-model organisms to the Gene Ontology database and molecular pathways such as KEGG pathways (62) is mainly based on the assumption that orthologous genes have similar functions in different species. In addition to genes present in many mammalian species, there are species-specific or group-specific genes that could get lost using our approach. However, many of these genes are of unknown function, thus not affecting the results of functional annotation analysis. Since Steps 2 and 3 of the MAdb approach can also deliver paralogs or genes that have moderate sequence similarity if no ortholog is present, some of these genes can be at least assigned to a gene which might have a similar function. Moreover, in case of the Gene Ontology database, except for the very specific functional categories, related genes are usually assigned to the same categories. Another problem of using ortholog information is species-specific gene duplications, e.g. related to adaptations to the environment or host-pathogen interactions which were found for domestic animals (63). These genes, if differentially expressed in the experimental model, are underrepresented in the corresponding functional category or pathway. Since many of these duplicated genes do not yet have an official gene symbol, they are not assigned to functional databases and it does not make a difference if using the original species gene ID or the corresponding human identifier.

The comparison of MAdb to similar ortholog databases showed a better or, in the case of Ensembl Compara, a similar performance. All other database sources provided much fewer ID pairs or even wrong assignments. The reasons are mainly different, smaller (incomplete) or less up-to-date data sources. In the case of NCBI gene2ensembl, filtering of overlapping gene annotations is much more stringent (45). Ensembl uses four mapping strategies, which are based on third-party ID mappers, location overlaps but also sequence matches and alignments using exonerate (64,65). Another important point is that Ensembl only uses a fraction (i.e. manually annotated messenger RNAs and proteins) of the NCBI Refseq database (66). This could explain why our approach results in more ID pairs. Using the OMABrowser, we found only a fraction of the known human orthologs, due to the ID mapping of the OMABrowser (Ensembl gene ID to Entrez Gene ID) (personal communication with the authors of the OMABrowser). In some cases (e.g. Entrez Gene ID: 100621538, histone H2A type 3) we found an assignment of many human potential orthologs to just one pig histone gene. This might be due to missing annotation information in the pig genome for the numerous very similar histone genes but should be handled by the ortholog database filters. By not filtering these one-to-many orthologs, the functional annotation is strongly biased. The OMABrowser is based on Ensembl and the identifier mapping is also based on Uniprot. However, when starting from the Ensembl gene IDs, the obtained DAVID results were comparable to MAdb. Using our ID mapper AOR to convert Entrez Gene IDs to Ensembl gene IDs and then retrieving human with the OMABrowser did reach the results of MAdb. Although MAdb revealed more identifier matches, the functional annotation results were similar to Ensembl. However, the analysis of the gene identifier matching between Ensembl and NCBI revealed a considerable number of wrong assignments in the Ensembl database indicating multiple hits in the results due to one-to-many assignments of Ensembl to NCBI genes. When starting from NCBI gene IDs, the wrong assignments in Ensembl will affect the results in two steps, first with the conversion of the original NCBI ID to the Ensembl ID and the conversion of the Ensembl ortholog gene ID, e.g. human, back to the NCBI ortholog gene ID. In contrast, the number of wrong assignments in MAdb is very low and it does not make a difference if the analysis starts from NCBI or Ensembl gene IDs. The incorrect assignments between Ensembl gene IDs and external references such as NCBI gene IDs are usually between genes of high sequence similarity. Therefore, this does not dramatically affect the functional annotation analysis results when NCBI gene IDs are used that were translated from Ensembl gene IDs based on the information about external references in Ensembl. But if more than one NCBI gene IDs are assigned to one Ensembl gene ID, the results are biased. Furthermore, a deeper analysis of genes found in overrepresented functional categories could be misleading if the NCBI gene ID does not correspond to the correct Ensembl gene.

Overall, although it seems simple to assign ortholog gene IDs in order to perform improved functional annotation analysis and to use ortholog information from different annotation sources, this approach showed unexpected complexity. First, the topic of gene orthology is very complex (67–69). For many genes, not only are there are one-to-one orthologs between mammalian species but also a considerable number of genes specific for a species or a respective clade of mammals exist. The presence of duplicated or on the other side inactivated genes results in a number of problems for the assignment of orthologous genes and affect subsequent overrepresentation analyses of functional categories and molecular pathways in multiple ways. Furthermore, functional annotation transfer between species is based on conserved functions of orthologous genes, which is not always the case (67). The use of information from different annotation resources also revealed a number of problems with respect to the correct assignment of annotated genes between the NCBI and Ensembl annotation pipeline. Even for the human genome that was first published in 2003 (70,71) where large full-length complementary DNA sequencing projects have been performed analyzing almost all human tissues to cover specific transcript isoforms, there is only minimal agreement about gene annotation regarding the various transcript isoforms and alternative exon sequences between NCBI and Ensembl (72). If the assignment of corresponding genes between two annotation sources is incomplete or contains errors, the information will be lost or wrong information, e.g. about ortholog relationships, could be obtained.

## Conclusions

In summary, our novel database MAdb represents a tool for researchers working on non-classical as well as the classical mammalian model organisms who are interested in (i) the improvement of functional annotation of lists of DEGs or DEPs, (ii) in cross-species comparisons of transcriptomics or proteomics datasets, (iii) in the correct assignment of gene IDs between NCBI and Ensembl and, furthermore, (iv) in obtaining additional annotation information to lists of gene IDs and RefSeq transcript and protein IDs. We showed the benefits of using the MAdb and the improvements of the more accurate and complete mapping of IDs from Ensembl to NCBI. In addition, the database provides the freedom to use NCBI or Ensembl gene IDs.

## Availability of data and materials

The AnnOverlappeR is an open source collaborative initiative available in the GitHub repository (https://github.com/ bimbam23/AnnOverlappeR.git). The MAdb web page is hosted at https://modb.ethz.ch.

## Supplementary Material

suppFig_with_pics_neweg_baz086

Supplemental_Figures_baz086
