# Supplementary material for: Mammalian Annotation Database for improved annotation and functional classification of Omics datasets from less well-annotated organisms
Source: Database (Oxford). 2019 Jul 26;2019:baz086. doi: 10.1093/database/baz086 (PMC6661403; doi:10.1093/database/baz086)
Supplement: suppFig_with_pics_neweg_baz086 [file suppfig_with_pics_neweg_baz086.docx]

**Supplementary Materials for:**

**A Mammalian Annotation Database for improved annotation and functional classification of Omics datasets from less well annotated organisms**

**Authors:** Jochen T. Bick^1^, Shuqin Zeng^1,2^, Mark D. Robinson^3^, Susanne E. Ulbrich^1^, Stefan Bauersachs^1,2,*^

^1^Animal Physiology, Institute of Agricultural Sciences, ETH Zurich, Zurich, Switzerland

^2^Current address: Genetics and Functional Genomics, Clinic of Reproductive Medicine, Department for Farm Animals, University of Zurich, Zurich, Switzerland

^3^Institute of Molecular Life Sciences and SIB Swiss Institute of Bioinformatics, University of Zurich, Zurich, Switzerland

*Correspondence to: stefan.bauersachs@uzh.ch

**This PDF file includes:**

Figures S1 to S14

Tables S1 and S2

Supplementary Figure S1. Assign
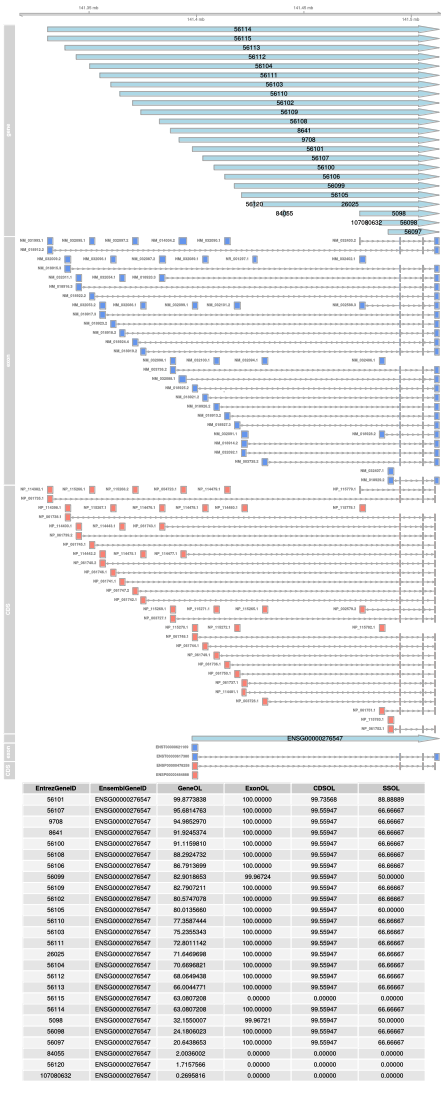
ment of the protocadherin gamma subfamily B, 6 (*PCDHGB6*) gene located in the protocadherin gamma gene cluster

In this example we present the annotation overlap of a gene cluster. Multiple genes have a high overlap on gene and exon or CDS level. The nomenclature for figures S1, S2 and S4 - S13 is identical: GeneOL = gene overlap, ExonOL = exon overlap, CDSOL = coding sequence overlap, SSOL = splice site overlap. This is one of the examples in which the duplication filter is important. In this case we find the best overlapping hit and consider it to be the only true positive identifier pair.

Supplementary Figure S2. Multiple overlaps

This example shows an overlap where both hits are higher than 50% but only the greater overlap is t
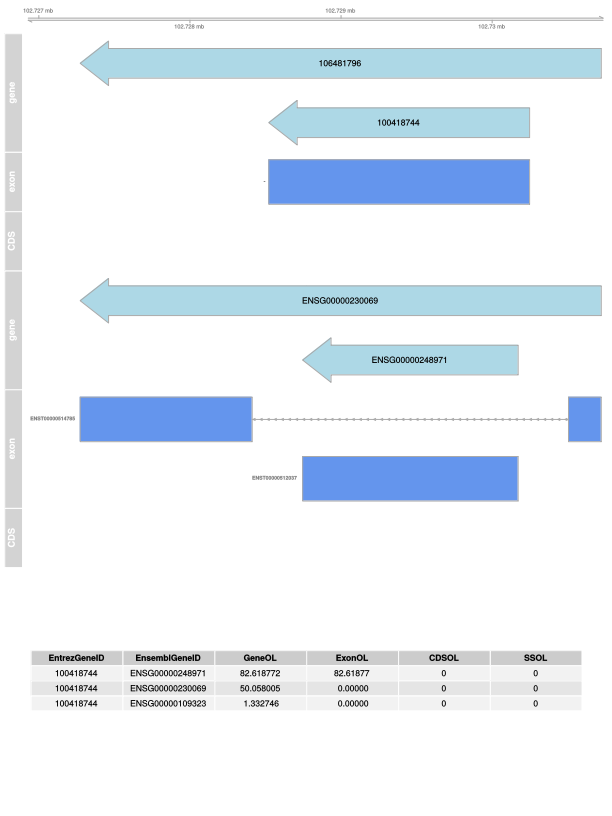
he correct or more reliable identifier pair. This example was also solved using the duplication filter.

Supplementary Figure S3. Gene overlap versus max(exon, CDS) overlap.

This scatter plot represents the distribution of gene overlap versus max(exon, CDS) overlap per i
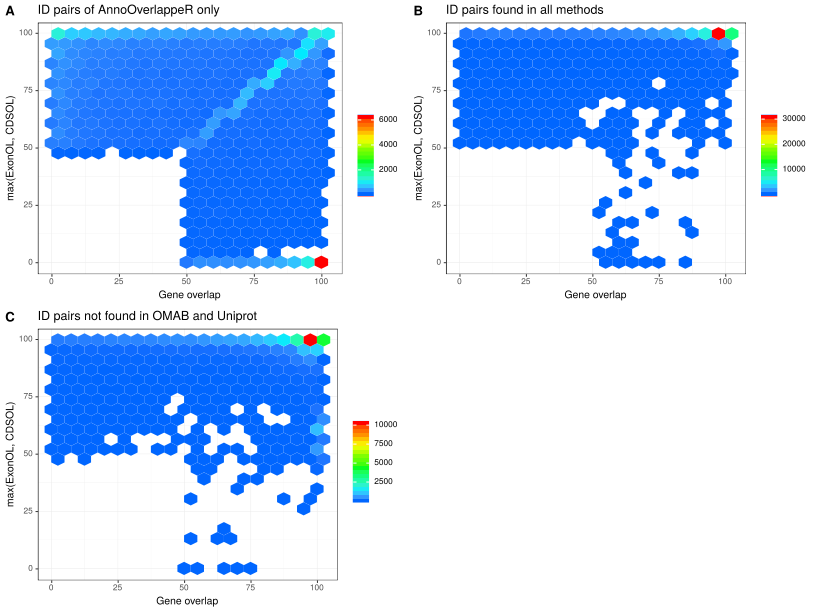
dentifier method (OMAB = OMABrowser)


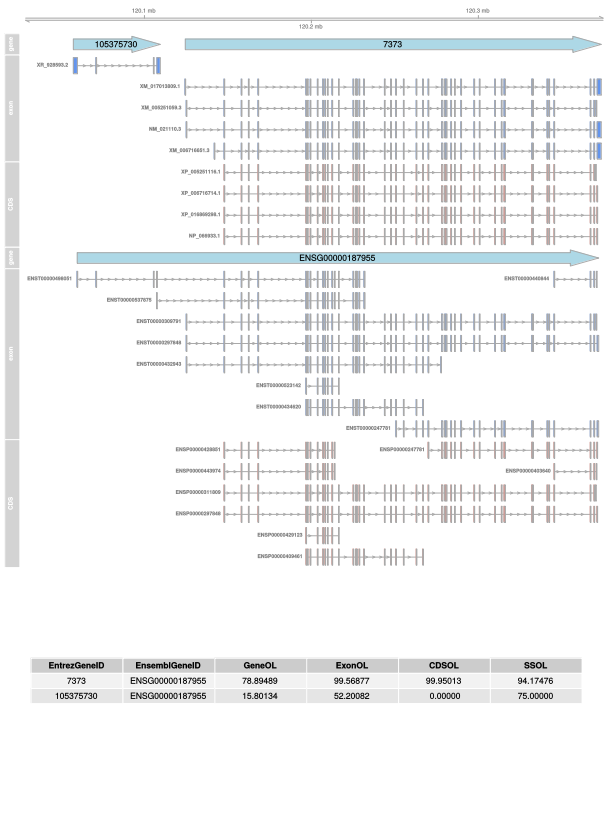
Supplementary Figure S4. One-to-many overlap example 1.This example shows that Ensembl and NCBI have a quite different annotation pipeline which can result in annotation of two (NCBI) genes instead of just one (Ensembl) in the same locus. Most of these cases had to be check manually to not get removed by the duplication filter.

Supplementary Figure S5. One-to-many overlap example 2.

Another example is shown here where the gene overlap is lower than 25% but the exon or/and CDS overlap is mostly higher than 50%. Here we show that there is a relatively high agreement between the annotation of NCBI and Ensembl at exon or CDS level but a difference in the definition of genes.


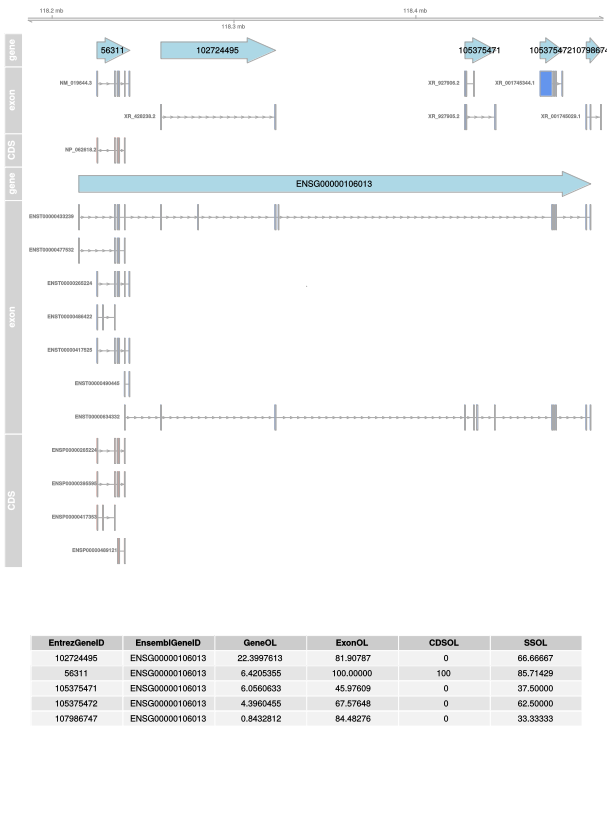


Supplementary Figure S6. Low gene overlap but identical exon and CDS overlap.

This example shows that due to additional untranslated exons (left) annotated at Ensembl the gene ENSG00000172757 is much longer than the gene 1072 at NCBI. Exon and CDS structures show
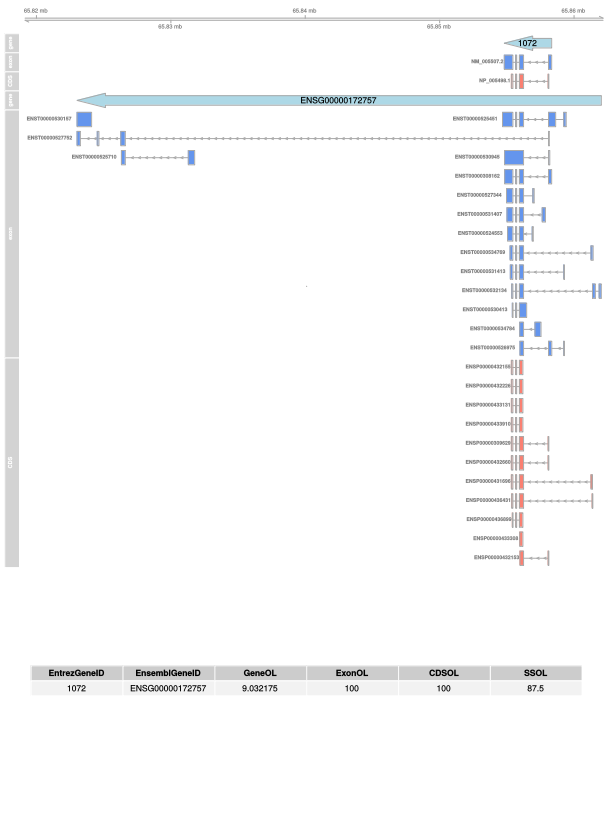
high overlap with respect to the NCBI gene.

Supplementary Figure S7. At the threshold of the overlap filter.

In this example, gene and exon or CDS overlap was too low to give evidence for a IDP according to t
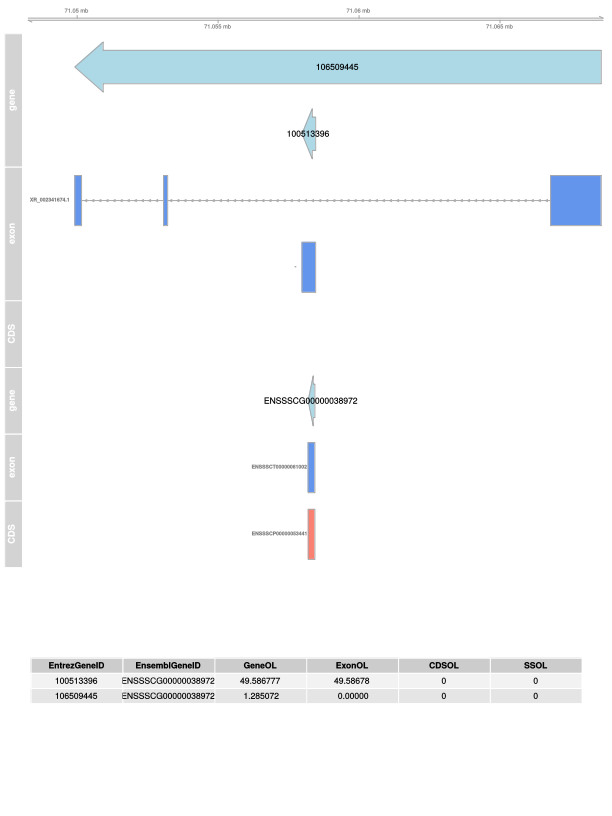
he threshold set at 50%.

Supplementary Figure S8. Problems with multiple IDs in the GFF.

This figure shows problems with multiple hit of the same ID in a GFF file. This might be a small
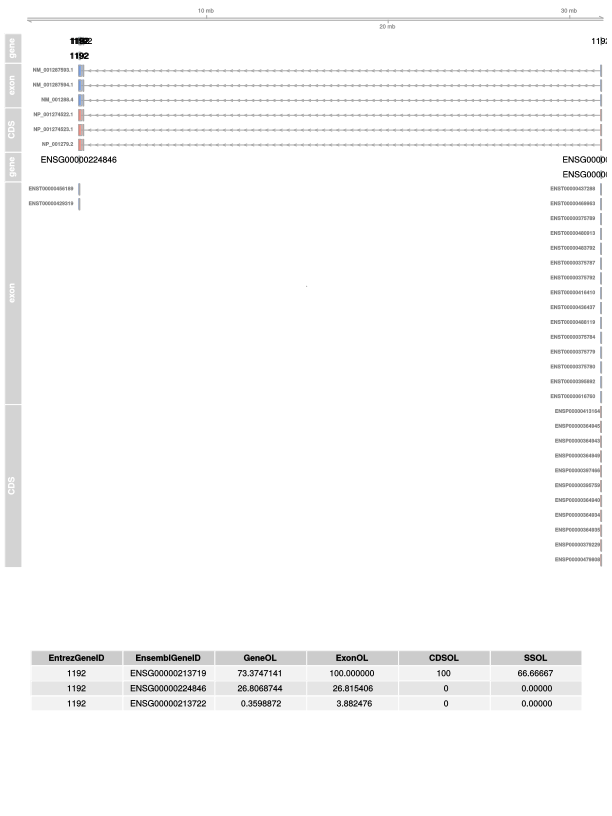
source of false positive which has to be handled manually.

**Supplementary Figure S9. Only the best hit.**

In this example we show that in some cases it is difficult to find the best hit of an ID pair. An exon or CDS overlap of 0% most likely means that either NCBI or Ensembl has no exon or CDS structure annotated so that there is no overlap possible.


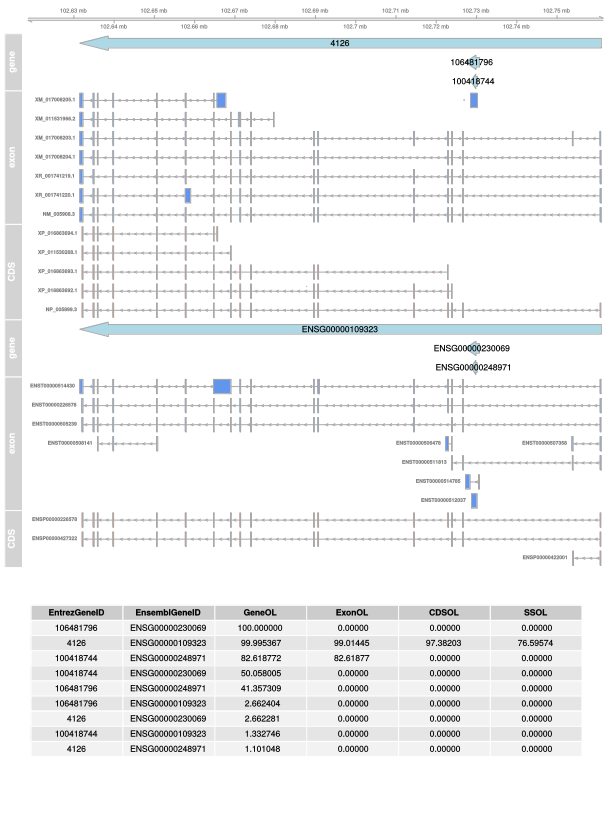


Supplementary Figure S10. Almost identical genes.

This figure shows a case where Ensembl annotated two almost identical gene. Most likely, both
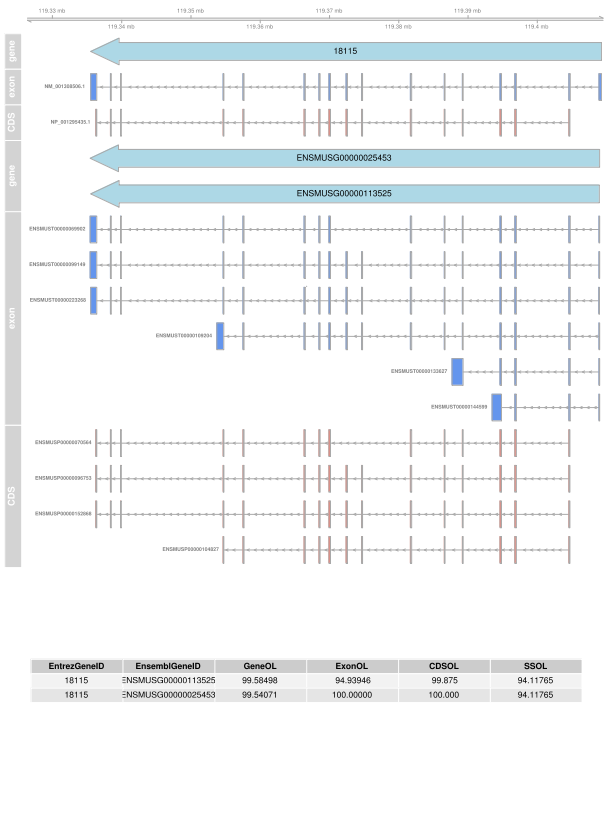
genes are pairing with one corresponding gene in NCBI. Manually checking/correcting was required.

Supplementary Figure S11. Only the best hit filter assigns the correct ID pair.


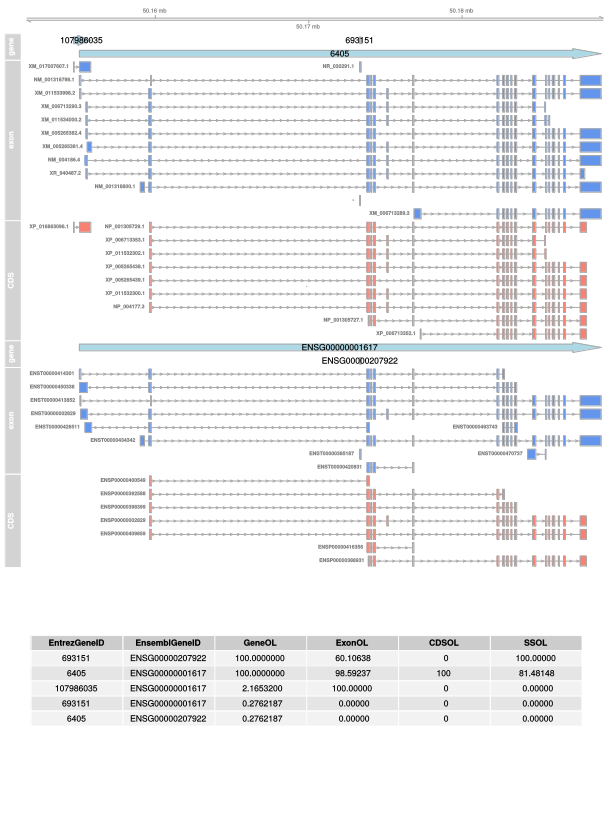
This figure shows again the problem of finding the right ID pair in case of nesting of multiple genes.

Supplementary Figure S12. Multiple overlapping genes.

This case shows that Ensembl sometimes annotated multiple genes to identical start positions. The
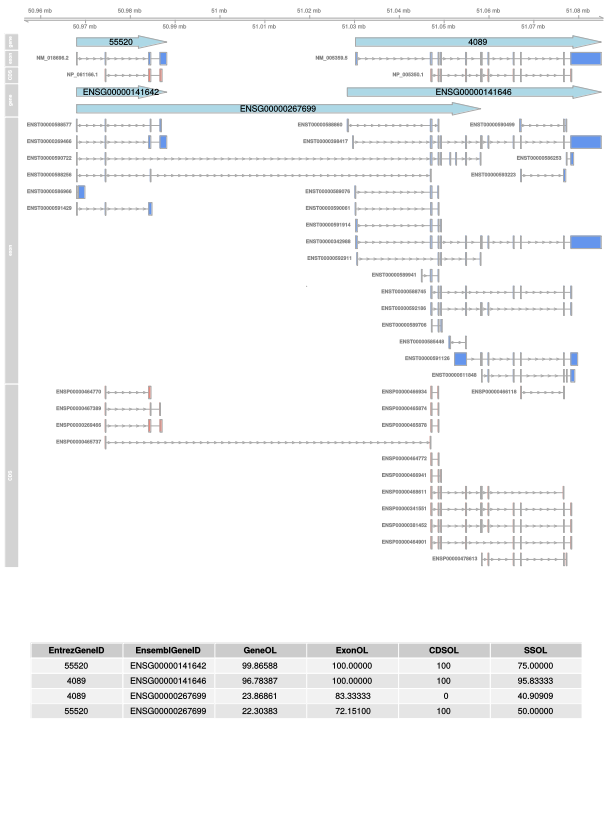
problem is again the correct assignment to NCBI, which was manually corrected.

Supplementary Figure S13. Analysis of missing identifier pairs (IDP).

These bar plots show reasons why AnnOverlappeR was not identifying some ID pairs found by other database sources.


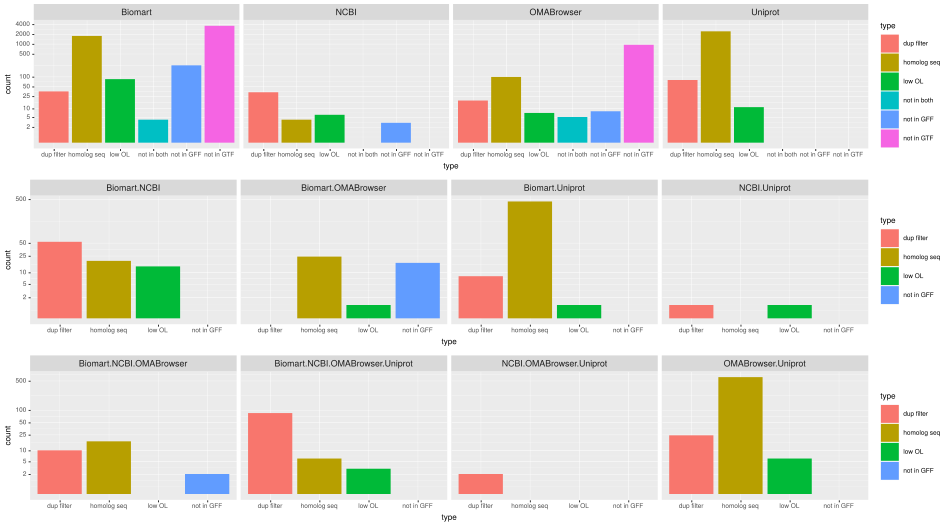


Supplementary Figure S14. Overlap of identified human orthologous genes for the test data set from the pig in comparison of 4 different database sources.

These 4 Venn diagrams show in **A** and **B** the overlapping detected human ortholog Entrez Gene IDs of 4 different database sources (EnsComp: Ensembl Compara, OMABAnnOL: OMABrowser plus AnnOverlappeR, OMABrowser, and MOAdb) separated by up- and down-regulated DEGs in porcine (pig) endometrium. Figure parts **C** and **D** represent the overlap of Ensembl gene IDs of
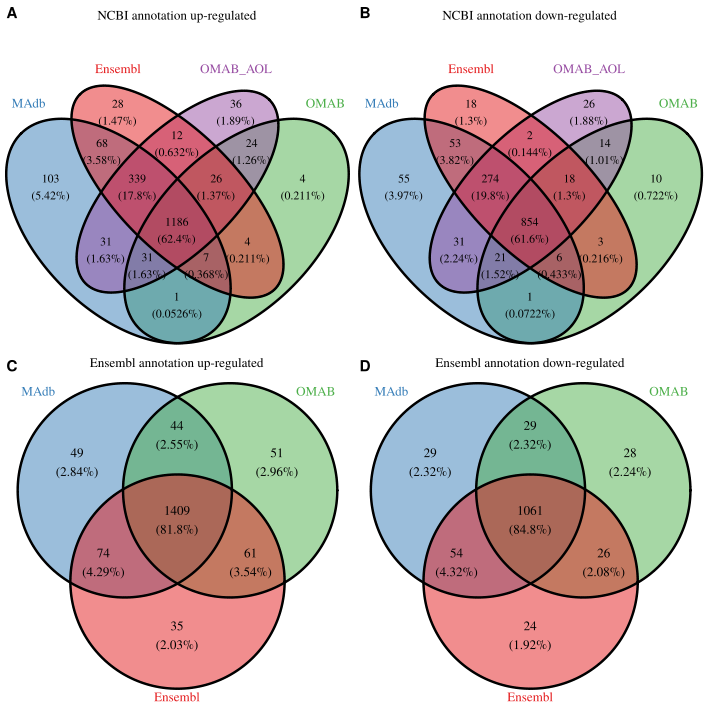
human orthologs of three database-sources also separated into up- and down-regulated DEGs.

**Table S1. BLASTn parameters.**

| **parameter** | **value** |
| --- | --- |
| -num_threads | 6 |
| -outfmt | 7 qseqid sseqid pident length mismatch gapopen qstart qend sstart send evalue bitscore qcovs |
| -dust | no |
| -gapopen | 1 |
| -gapextend | 1 |
| -penalty | -2 |
| -reward | 1 |
| -evalue | 1.00E-20 |
| -word_size | 7 |
| -max_target_seqs | 1 |
| -task | blastn |
| -strand | plus |

**Table S2. Number of gene for all seven species per type of gene.**

The first table shows gene information from the NCBI (GENE_INFO), the second table shows all genes with at least one ortholog retrieved from MAdb. The third table represents the percentage of genes that have an ortholog in MAdb.

| **GENE_INFO** | **protein-coding** | **ncRNA** | **pseudo** | **other** | **tRNA** | **rRNA** | **scRNA** | **snoRNA** | **snRNA** | **unknown** | **biological-region** | **sum** |
| --- | --- | --- | --- | --- | --- | --- | --- | --- | --- | --- | --- | --- |
| bos taurus | 23134 | 6062 | 5000 | 259 | 1638 | 21 | 0 | 634 | 978 | 20058 | 0 | 57784 |
| canis familiaris | 20422 | 11325 | 5379 | 170 | 397 | 0 | 0 | 0 | 0 | 8 | 0 | 37701 |
| equus caballus | 21498 | 7551 | 2858 | 279 | 474 | 12 | 0 | 447 | 432 | 20 | 0 | 33571 |
| homo sapiens | 20214 | 17276 | 16436 | 855 | 599 | 59 | 4 | 541 | 64 | 1770 | 3625 | 61443 |
| mus musculus | 27333 | 15412 | 10758 | 2648 | 523 | 47 | 13 | 132 | 20 | 10530 | 751 | 68167 |
| oryctolagus cuniculus | 20803 | 3943 | 5382 | 250 | 485 | 3 | 0 | 3 | 0 | 4 | 0 | 30873 |
| sus scrofa | 21512 | 6061 | 3090 | 142 | 510 | 2 | 0 | 0 | 0 | 14787 | 0 | 46104 |
|  |  |  |  |  |  |  |  |  |  |  |  |  |
| **Ortholog hit found in MOAdb** | **protein-coding** | **ncRNA** | **pseudo** | **other** | **tRNA** | **rRNA** | **scRNA** | **snoRNA** | **snRNA** | **unknown** | **biological-region** | **sum** |
| bos taurus | 20033 | 621 | 687 | 89 | 0 | 5 | 0 | 473 | 609 | 50 | 0 | 22567 |
| canis familiaris | 18947 | 539 | 592 | 90 | 0 | 0 | 0 | 0 | 0 | 4 | 0 | 20172 |
| equus caballus | 20215 | 620 | 345 | 180 | 0 | 9 | 0 | 394 | 350 | 15 | 0 | 22128 |
| homo sapiens | 19099 | 1008 | 1050 | 333 | 0 | 15 | 2 | 374 | 28 | 7 | 0 | 21916 |
| mus musculus | 20434 | 651 | 292 | 463 | 0 | 16 | 2 | 123 | 12 | 36 | 0 | 22029 |
| oryctolagus cuniculus | 18807 | 131 | 718 | 156 | 0 | 1 | 0 | 3 | 0 | 1 | 0 | 19817 |
| sus scrofa | 19782 | 515 | 940 | 123 | 0 | 2 | 0 | 0 | 0 | 5 | 0 | 21367 |
|  |  |  |  |  |  |  |  |  |  |  |  |  |
| **percent** | **protein-coding** | **ncRNA** | **pseudo** | **other** | **tRNA** | **rRNA** | **scRNA** | **snoRNA** | **snRNA** | **unknown** | **biological-region** | **-** |
| bos taurus | 86.6 | 10.2 | 13.7 | 34.4 | 0.0 | 23.8 | 0.0 | 74.6 | 62.3 | 0.2 | 0.0 | - |
| canis familiaris | 92.8 | 4.8 | 11.0 | 52.9 | 0.0 | 0.0 | 0.0 | 0.0 | 0.0 | 50.0 | 0.0 | - |
| equus caballus | 94.0 | 8.2 | 12.1 | 64.5 | 0.0 | 75.0 | 0.0 | 88.1 | 81.0 | 75.0 | 0.0 | - |
| homo sapiens | 94.5 | 5.8 | 6.4 | 38.9 | 0.0 | 25.4 | 50.0 | 69.1 | 43.8 | 0.4 | 0.0 | - |
| mus musculus | 74.8 | 4.2 | 2.7 | 17.5 | 0.0 | 34.0 | 15.4 | 93.2 | 60.0 | 0.3 | 0.0 | - |
| oryctolagus cuniculus | 90.4 | 3.3 | 13.3 | 62.4 | 0.0 | 33.3 | 0.0 | 100.0 | 0.0 | 25.0 | 0.0 | - |
| sus scrofa | 92.0 | 8.5 | 30.4 | 86.6 | 0.0 | 100.0 | 0.0 | 0.0 | 0.0 | 0.0 | 0.0 | - |
|  |  |  |  |  |  |  |  |  |  |  |  |  |
| average | 89.3 | 6.4 | 12.8 | 51.0 | 0.0 | 41.7 | 9.3 | 60.7 | 35.3 | 21.6 | 0.0 | - |
