# Supplementary material for: Mammalian Annotation Database for improved annotation and functional classification of Omics datasets from less well-annotated organisms
Source: Database (Oxford). 2019 Jul 26;2019:baz086. doi: 10.1093/database/baz086 (PMC6661403; doi:10.1093/database/baz086)
Supplement: Supplemental_Figures_baz086 [file supplemental_figures_baz086.pdf]

Supplementary Materials for:

A Mammalian Database for improved annotation and functional classification of Omics datasets from less well annotated organisms

Authors: Jochen T. Bick[1], Shuqin Zeng[1,2], Mark D. Robinson[3], Susanne E. Ulbrich[1], Stefan Bauersachs[1,2,*]

[1]Animal Physiology, Institute of Agricultural Sciences, ETH Zurich, Zurich, Switzerland

[2]Current address: Genetics and Functional Genomics, Vetsuisse Faculty Zurich, University of Zurich, Zurich, Switzerland

[3]Institute of Molecular Life Sciences and SIB Swiss Institute of Bioinformatics, University of Zurich, Zurich, Switzerland

*Correspondence to: stefan.bauersachs@uzh.ch

This PDF file includes:

Figures S1 to S14
Tables S1 and S2

Supplementary Figure S1. Assignment of the protocadherin gamma subfamily B, 6 (*PCDHGB6*) gene located in the protocadherin gamma gene cluster

In this example we present the annotation overlap of a gene cluster. Multiple genes have a high overlap on gene and exon or CDS level. The nomenclature for figures S1, S2 and S4 - S13 is identical: GeneOL = gene overlap, ExonOL = exon overlap, CDSOL = coding sequence overlap, SSOL = splice site overlap. This is one of the examples in which the duplication filter is important. In this case we find the best overlapping hit and consider it to be the only true positive identifier pair.

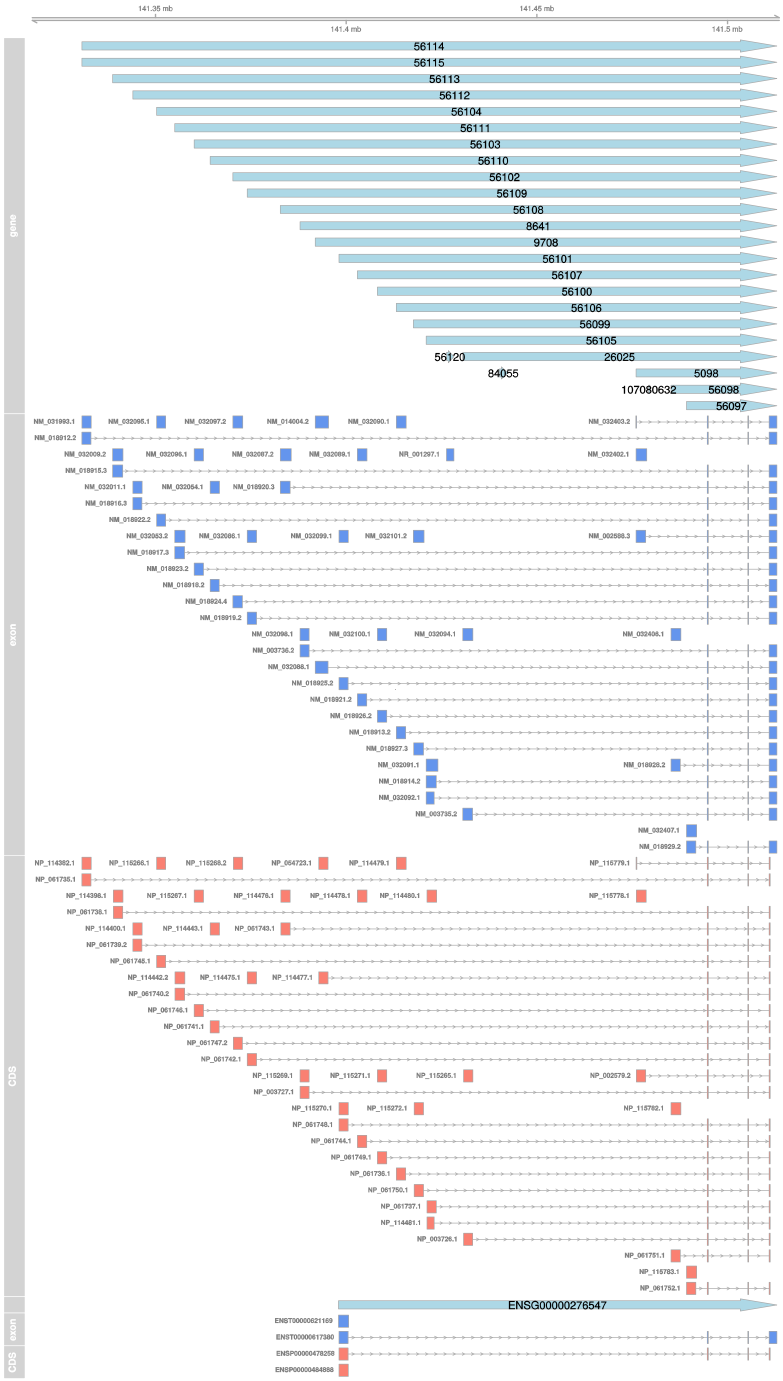

EntrezGeneID	EnsemblGeneID	GeneOL	ExonOL	CDSOL	SSOL
56101	ENSG00000276547	99.8773838	100.00000	99.73568	88.88889
56107	ENSG00000276547	95.6814763	100.00000	99.55947	66.66667
9708	ENSG00000276547	94.9852970	100.00000	99.55947	66.66667
8641	ENSG00000276547	91.9245374	100.00000	99.55947	66.66667
56100	ENSG00000276547	91.1159810	100.00000	99.55947	66.66667
56108	ENSG00000276547	88.2924732	100.00000	99.55947	66.66667
56106	ENSG00000276547	86.7913699	100.00000	99.55947	66.66667
56099	ENSG00000276547	82.9018653	99.96724	99.55947	50.00000
56109	ENSG00000276547	82.7907211	100.00000	99.55947	66.66667
56102	ENSG00000276547	80.5747078	100.00000	99.55947	66.66667
56105	ENSG00000276547	80.0135660	100.00000	99.55947	60.00000
56110	ENSG00000276547	77.3587444	100.00000	99.55947	66.66667
56103	ENSG00000276547	75.2355343	100.00000	99.55947	66.66667
56111	ENSG00000276547	72.8011142	100.00000	99.55947	66.66667
26025	ENSG00000276547	71.6469698	100.00000	99.55947	66.66667
56104	ENSG00000276547	70.6696821	100.00000	99.55947	66.66667
56112	ENSG00000276547	68.0649438	100.00000	99.55947	66.66667
56113	ENSG00000276547	66.0044771	100.00000	99.55947	66.66667
56115	ENSG00000276547	63.0807208	0.00000	0.00000	0.00000
56114	ENSG00000276547	63.0807208	100.00000	99.55947	66.66667
5098	ENSG00000276547	32.1550007	99.96721	99.55947	50.00000
56098	ENSG00000276547	24.1806023	100.00000	99.55947	66.66667
56097	ENSG00000276547	20.6438653	100.00000	99.55947	66.66667
84055	ENSG00000276547	2.0036002	0.00000	0.00000	0.00000
56120	ENSG00000276547	1.7157566	0.00000	0.00000	0.00000
107080632	ENSG00000276547	0.2695816	0.00000	0.00000	0.00000

Supplementary Figure S2. Multiple overlaps

This example shows an overlap where both hits are higher than 50% but only the greater overlap is the correct or more reliable identifier pair. This example was also solved using the duplication filter.

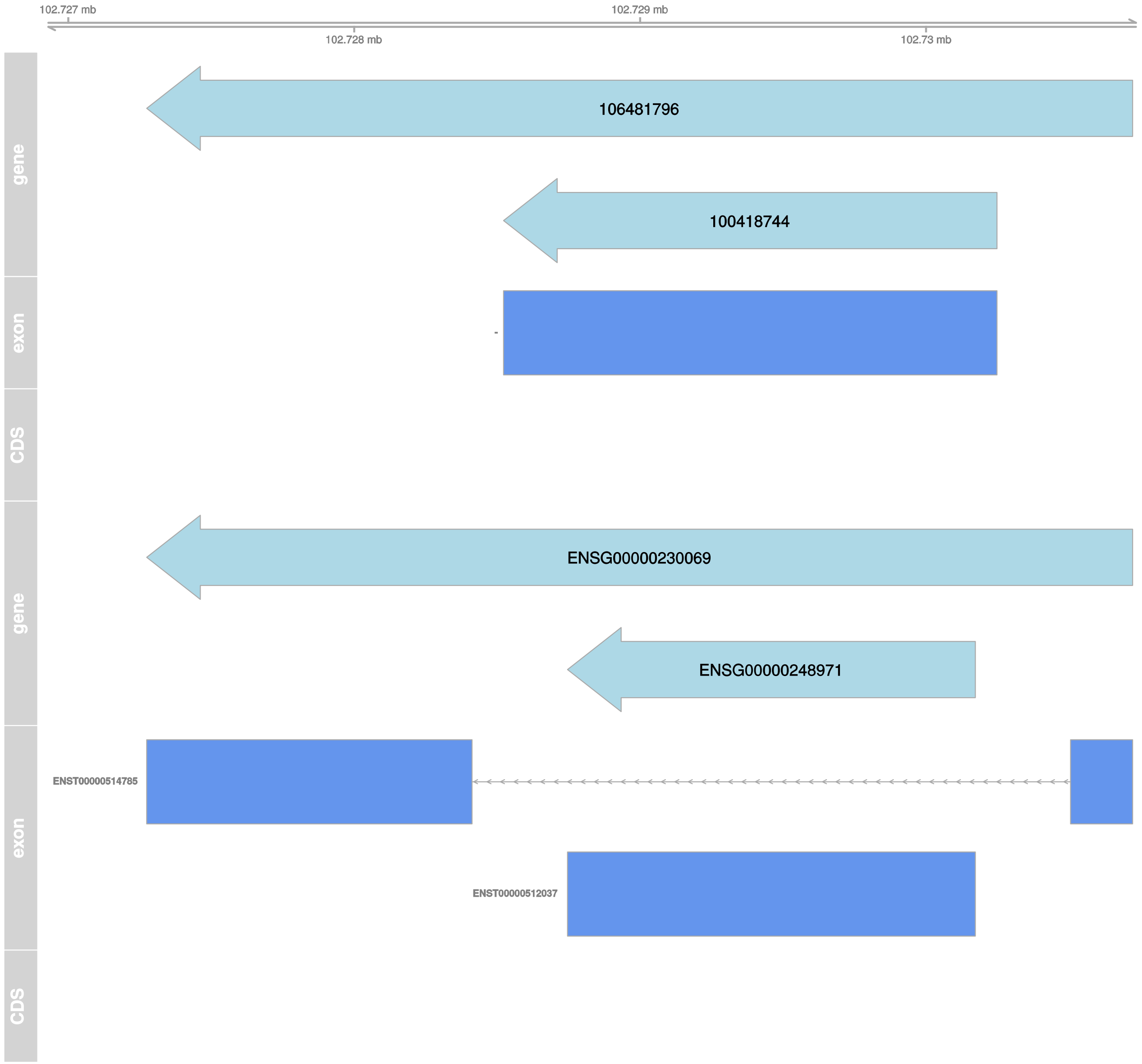

EntrezGeneID	EnsemblGeneID	GeneOL	ExonOL	CDSOL	SSOL
100418744	ENSG00000248971	82.618772	82.61877	0	0
100418744	ENSG00000230069	50.058005	0.00000	0	0
100418744	ENSG00000109323	1.332746	0.00000	0	0

Supplementary Figure S3. Gene overlap versus max(exon, CDS) overlap.

This scatter plot represents the distribution of gene overlap versus max(exon, CDS) overlap per identifier method (OMAB = OMABrowser)

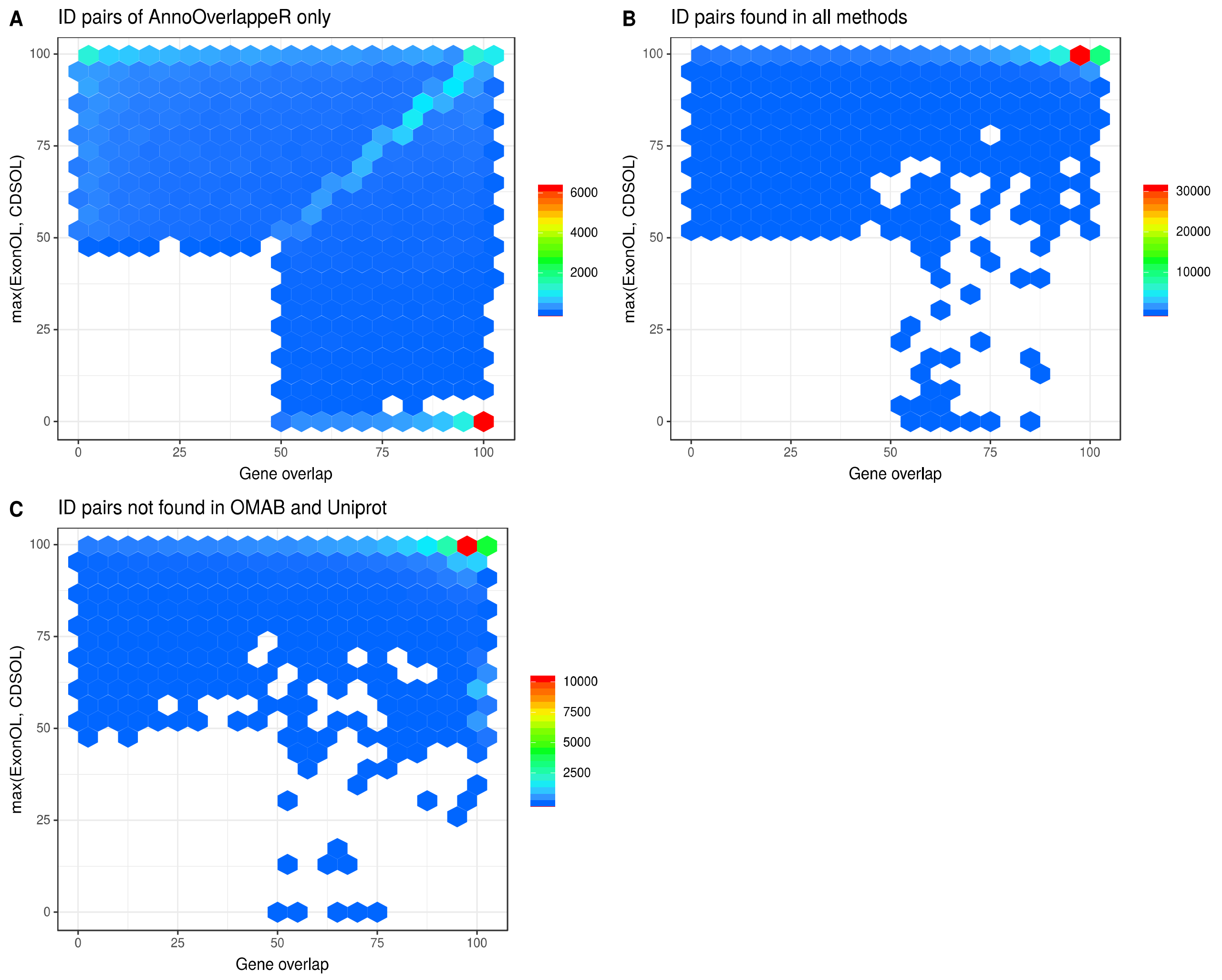

Supplementary Figure S4. One-to-many overlap example 1. This example shows that Ensembl and NCBI have a quite different annotation pipeline which can result in annotation of two (NCBI) genes instead of just one (Ensembl) in the same locus. Most of these cases had to be check manually to not get removed by the duplication filter.

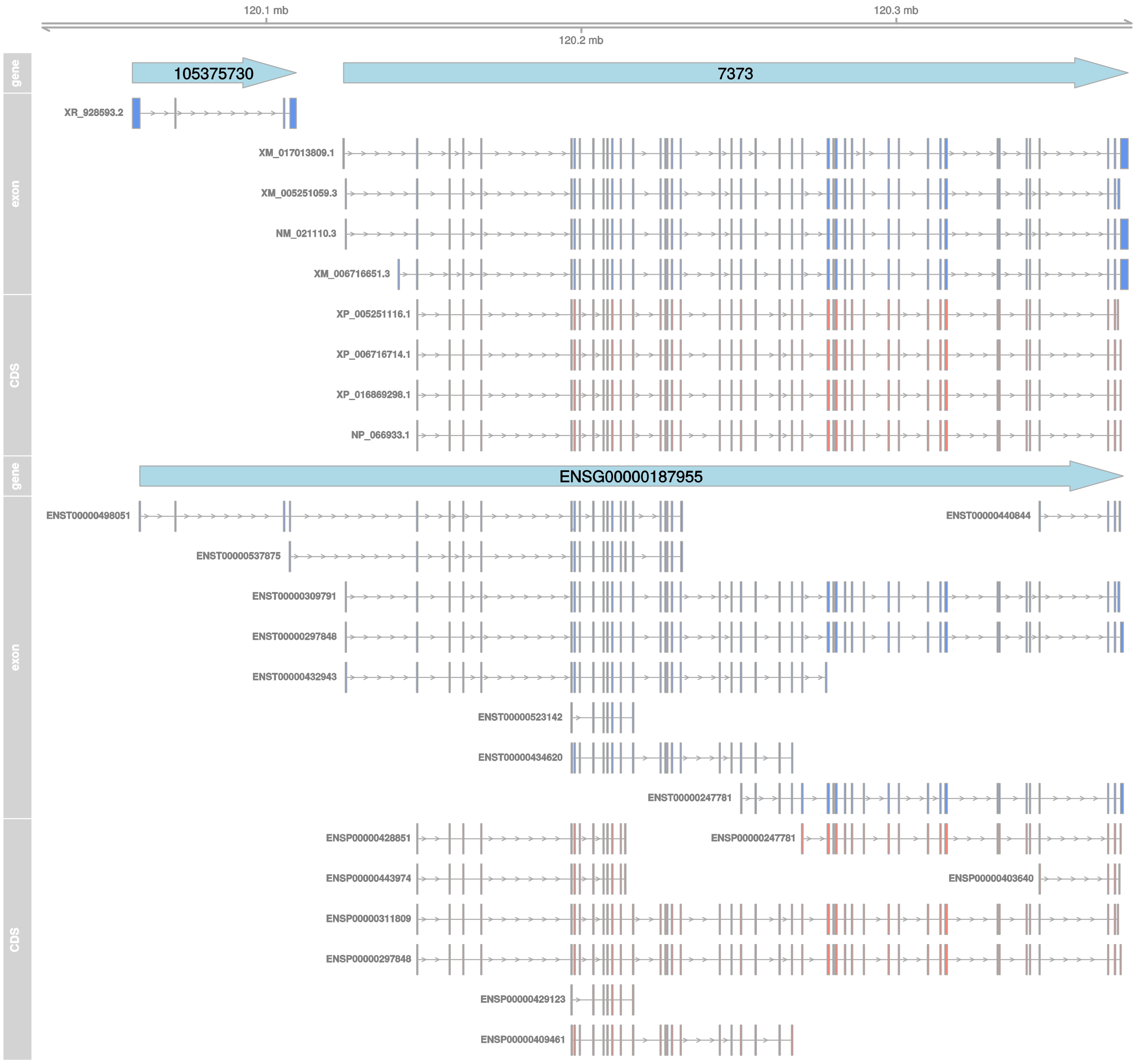

EntrezGeneID	EnsemblGeneID	GeneOL	ExonOL	CDSOL	SSOL
7373	ENSG00000187955	78.89489	99.56877	99.95013	94.17476
105375730	ENSG00000187955	15.80134	52.20082	0.00000	75.00000

Supplementary Figure S5. One-to-many overlap example 2.

Another example is shown here where the gene overlap is lower than 25% but the exon or/and CDS overlap is mostly higher than 50%. Here we show that there is a relatively high agreement between the annotation of NCBI and Ensembl at exon or CDS level but a difference in the definition of genes.

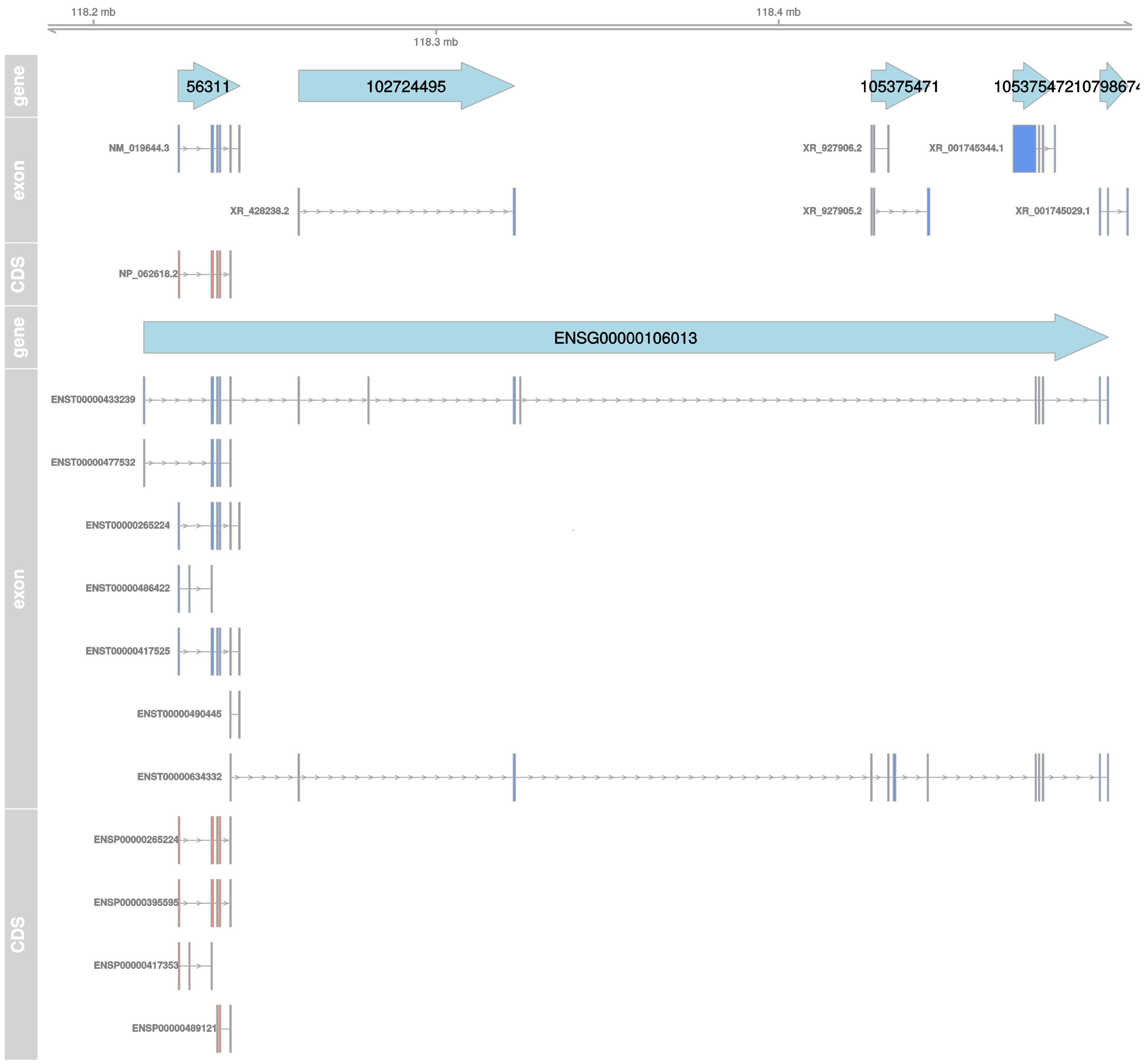

EntrezGeneID	EnsemblGeneID	GeneOL	ExonOL	CDSOL	SSOL
102724495	ENSG00000106013	22.3997613	81.90787	0	66.66667
56311	ENSG00000106013	6.4205355	100.00000	100	85.71429
105375471	ENSG00000106013	6.0560633	45.97609	0	37.50000
105375472	ENSG00000106013	4.3960455	67.57648	0	62.50000
107986747	ENSG00000106013	0.8432812	84.48276	0	33.33333

Supplementary Figure S6. Low gene overlap but identical exon and CDS overlap.

This example shows that due to additional untranslated exons (left) annotated at Ensembl the gene ENSG00000172757 is much longer than the gene 1072 at NCBI. Exon and CDS structures show high overlap with respect to the NCBI gene.

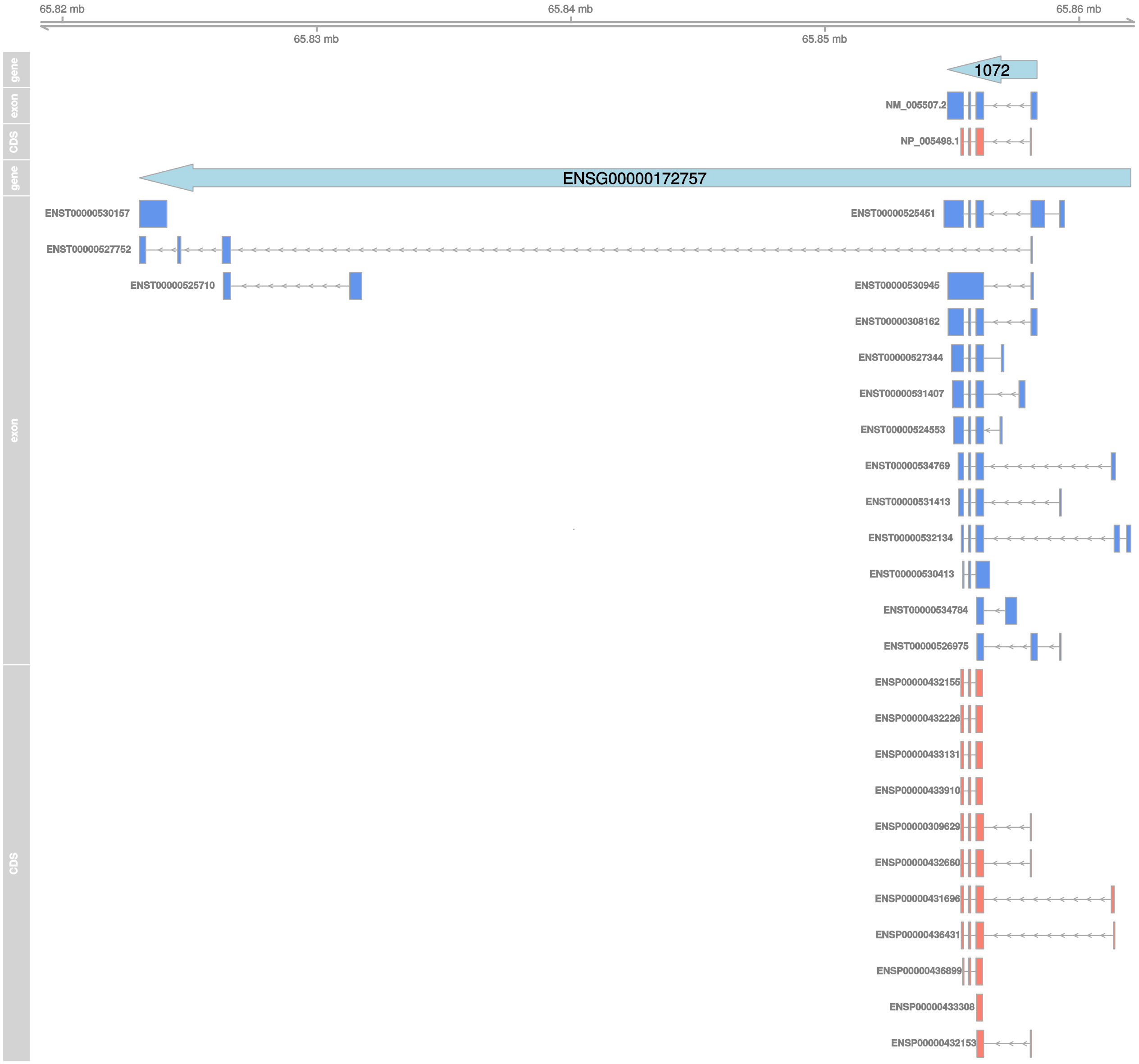

EntrezGeneID	EnsemblGeneID	GeneOL	ExonOL	CDSOL	SSOL
1072	ENSG00000172757	9.032175	100	100	87.5

Supplementary Figure S7. At the threshold of the overlap filter.

In this example, gene and exon or CDS overlap was too low to give evidence for a IDP according to the threshold set at 50%.

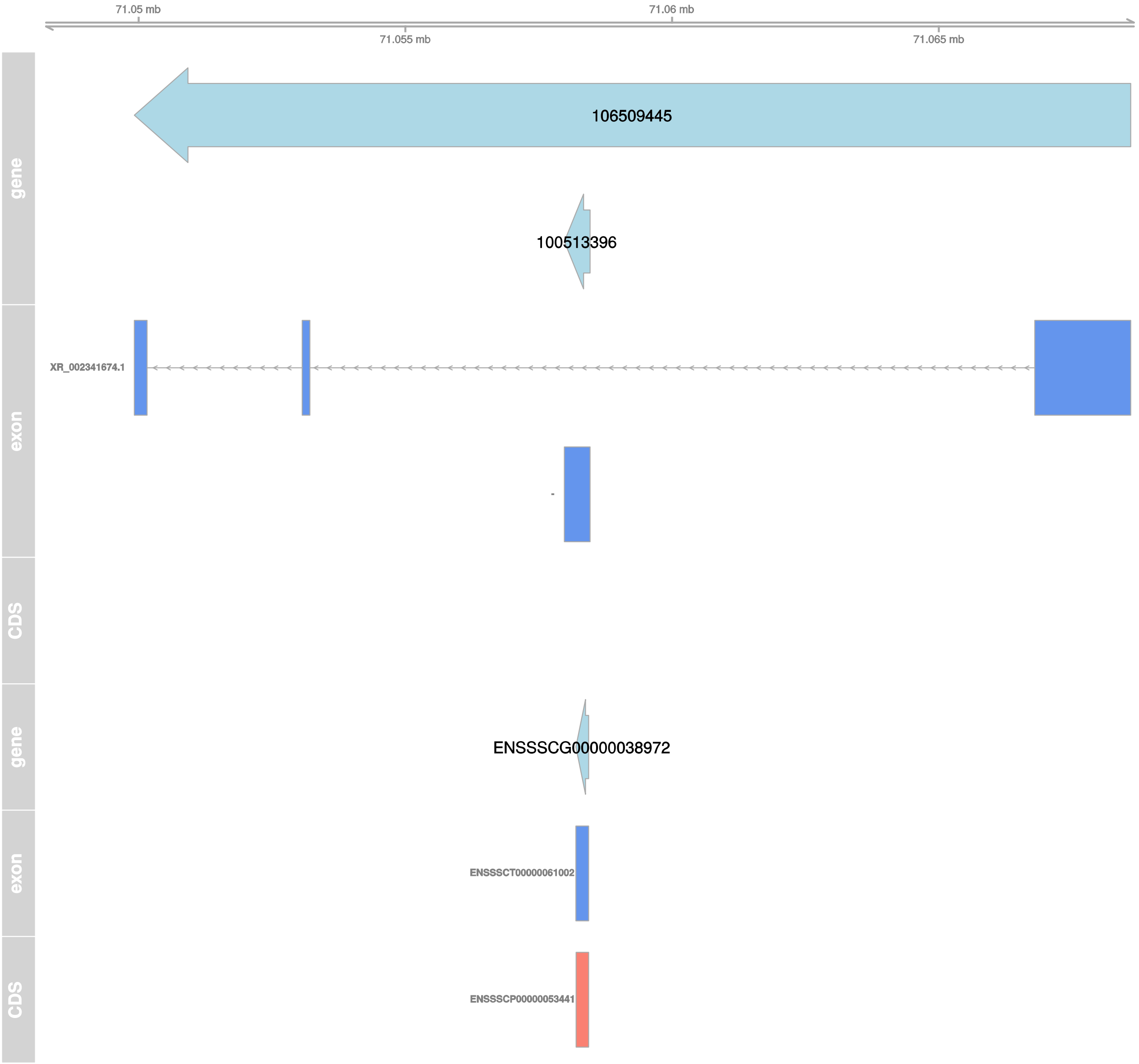

EntrezGeneID	EnsemblGeneID	GeneOL	ExonOL	CDSOL	SSOL
100513396	ENSSSCG00000038972	49.586777	49.58678	0	0
106509445	ENSSSCG00000038972	1.285072	0.00000	0	0

Supplementary Figure S8. Problems with multiple IDs in the GFF.

This figure shows problems with multiple hit of the same ID in a GFF file. This might be a small source of false positive which has to be handled manually.

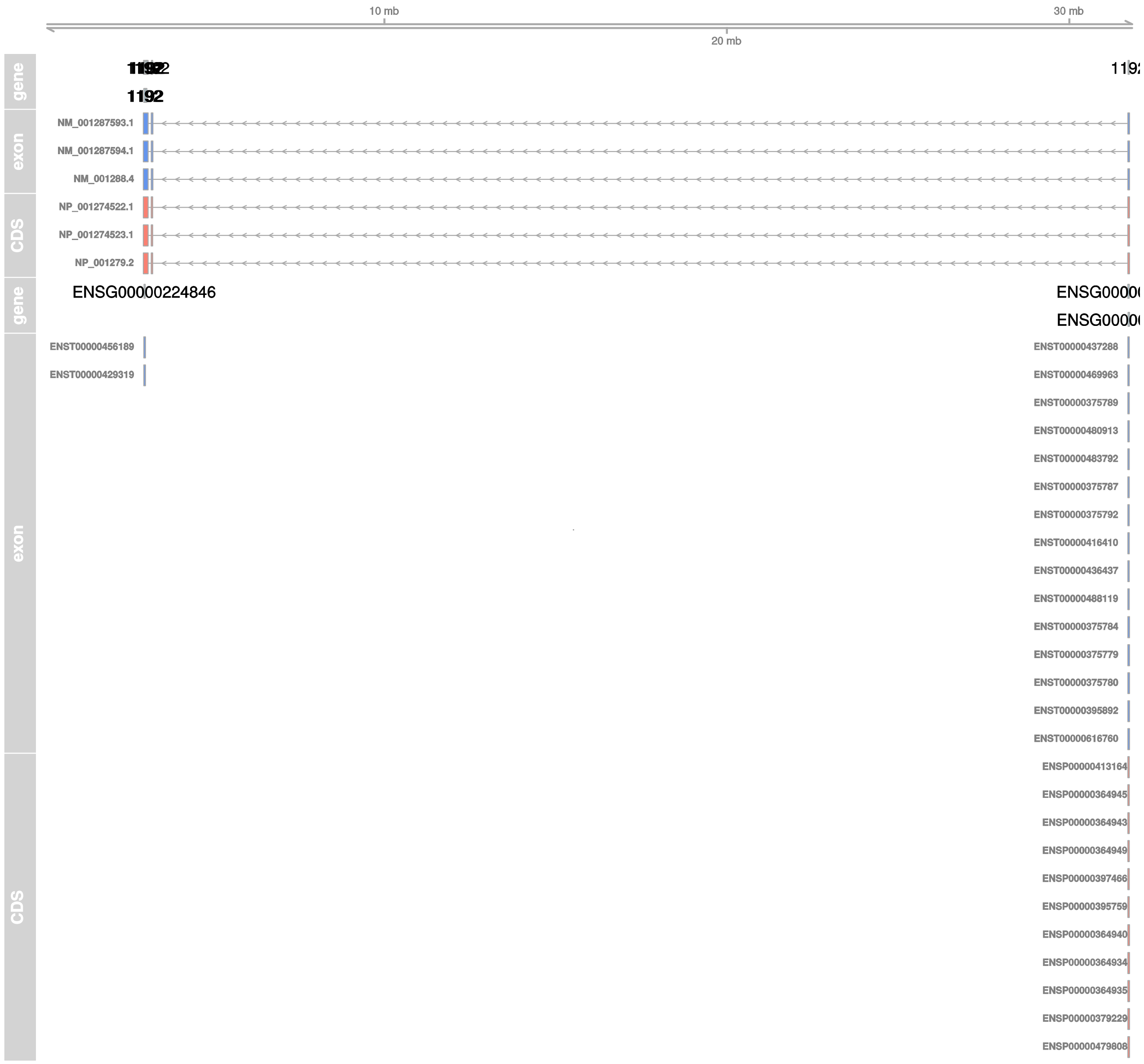

EntrezGeneID	EnsemblGeneID	GeneOL	ExonOL	CDSOL	SSOL
1192	ENSG00000213719	73.3747141	100.000000	100	66.66667
1192	ENSG00000224846	26.8068744	26.815406	0	0.00000
1192	ENSG00000213722	0.3598872	3.882476	0	0.00000

Supplementary Figure S9. Only the best hit.

In this example we show that in some cases it is difficult to find the best hit of an ID pair. An exon or CDS overlap of 0% most likely means that either NCBI or Ensembl has no exon or CDS structure annotated so that there is no overlap possible.

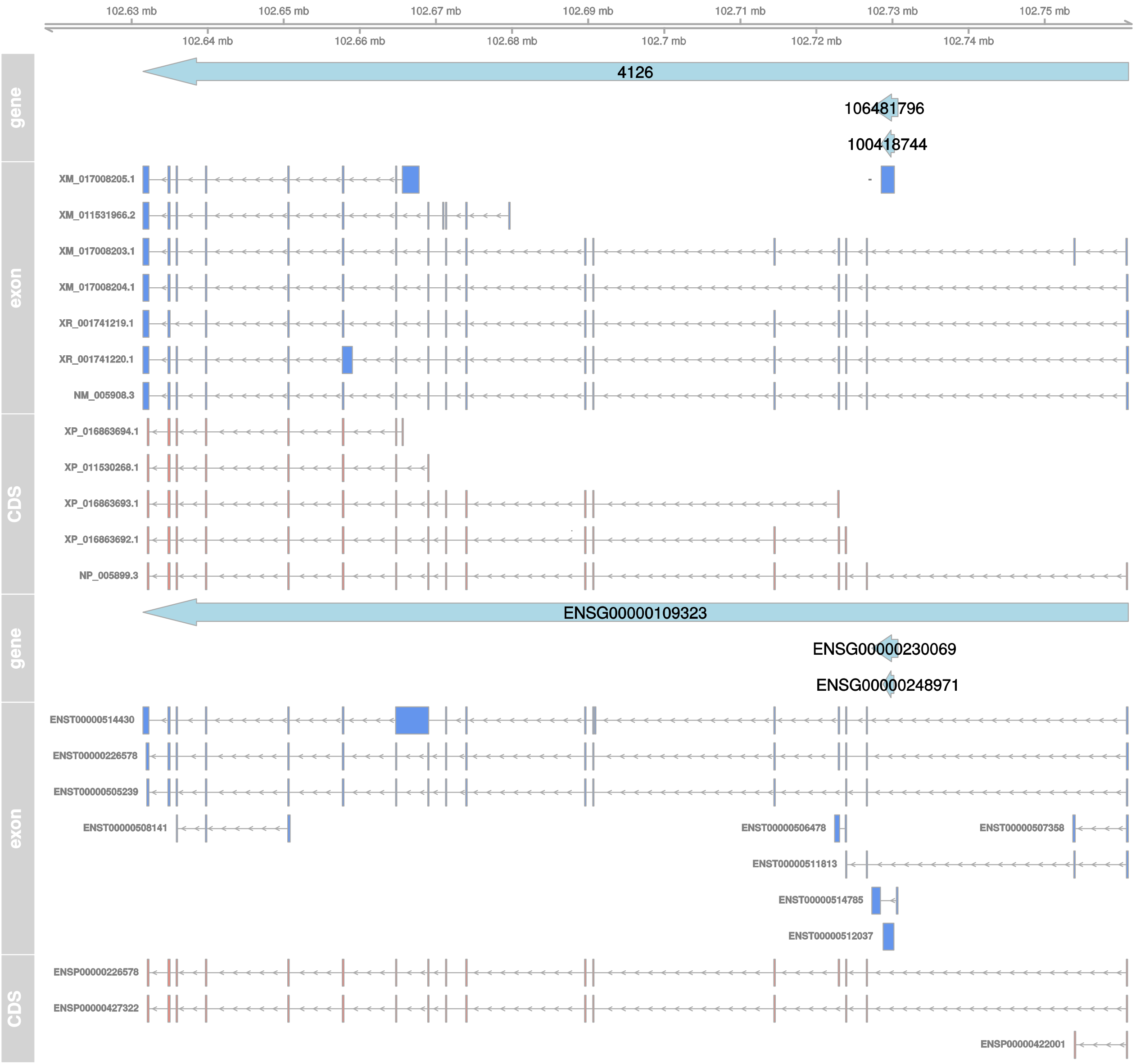

EntrezGeneID	EnsemblGeneID	GeneOL	ExonOL	CDSOL	SSOL
106481796	ENSG00000230069	100.000000	0.00000	0.00000	0.00000
4126	ENSG00000109323	99.995367	99.01445	97.38203	76.59574
100418744	ENSG00000248971	82.618772	82.61877	0.00000	0.00000
100418744	ENSG00000230069	50.058005	0.00000	0.00000	0.00000
106481796	ENSG00000248971	41.357309	0.00000	0.00000	0.00000
106481796	ENSG00000109323	2.662404	0.00000	0.00000	0.00000
4126	ENSG00000230069	2.662281	0.00000	0.00000	0.00000
100418744	ENSG00000109323	1.332746	0.00000	0.00000	0.00000
4126	ENSG00000248971	1.101048	0.00000	0.00000	0.00000

Supplementary Figure S10. Almost identical genes.

This figure shows a case where Ensembl annotated two almost identical gene. Most likely, both genes are pairing with one corresponding gene in NCBI. Manually checking/correcting was required.

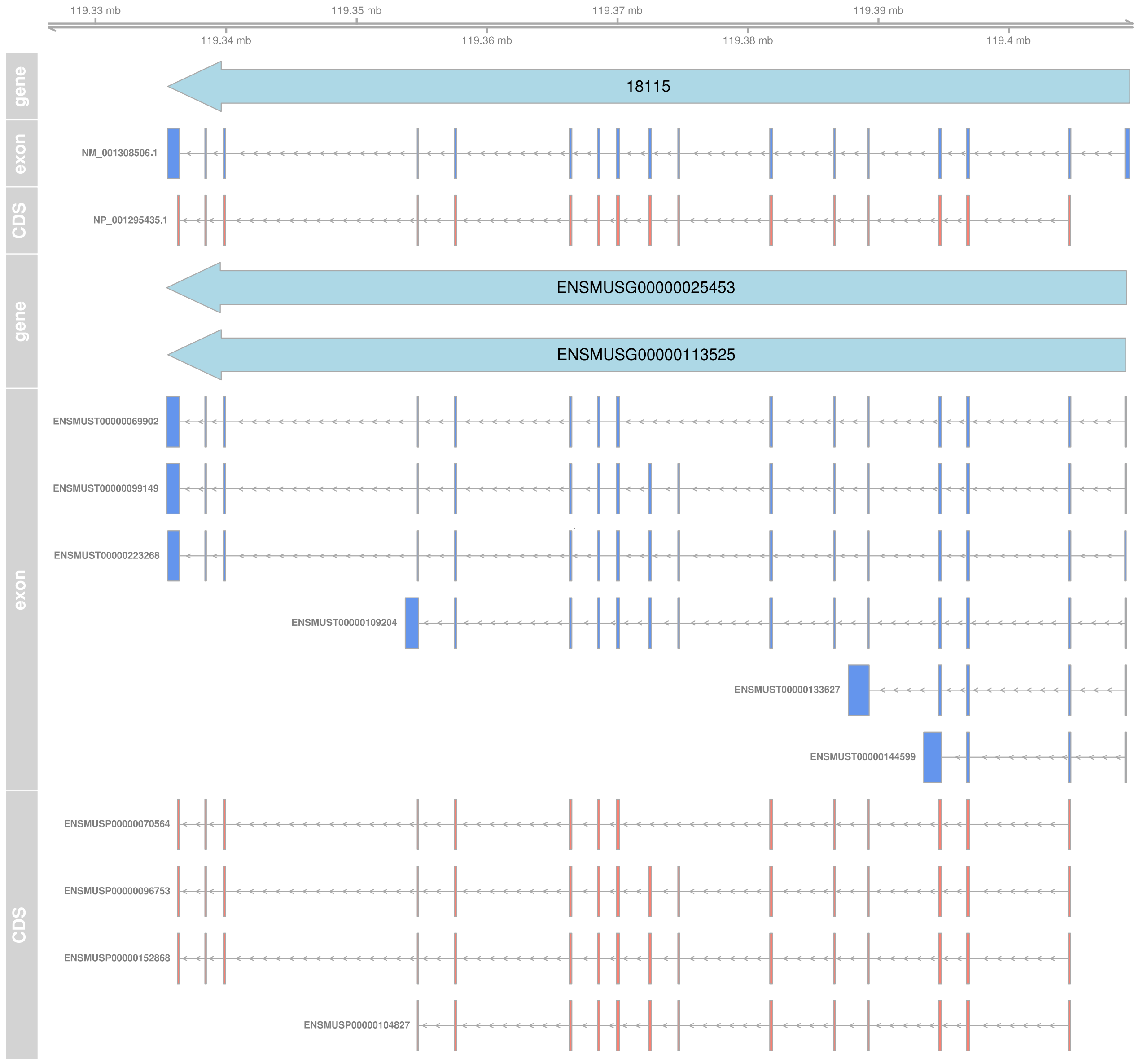

EntrezGeneID	EnsemblGeneID	GeneOL	ExonOL	CDSOL	SSOL
18115	ENSMUSG00000113525	99.58498	94.93946	99.875	94.11765
18115	ENSMUSG00000025453	99.54071	100.00000	100.000	94.11765

Supplementary Figure S11. Only the best hit filter assigns the correct ID pair.

This figure shows again the problem of finding the right ID pair in case of nesting of multiple genes.

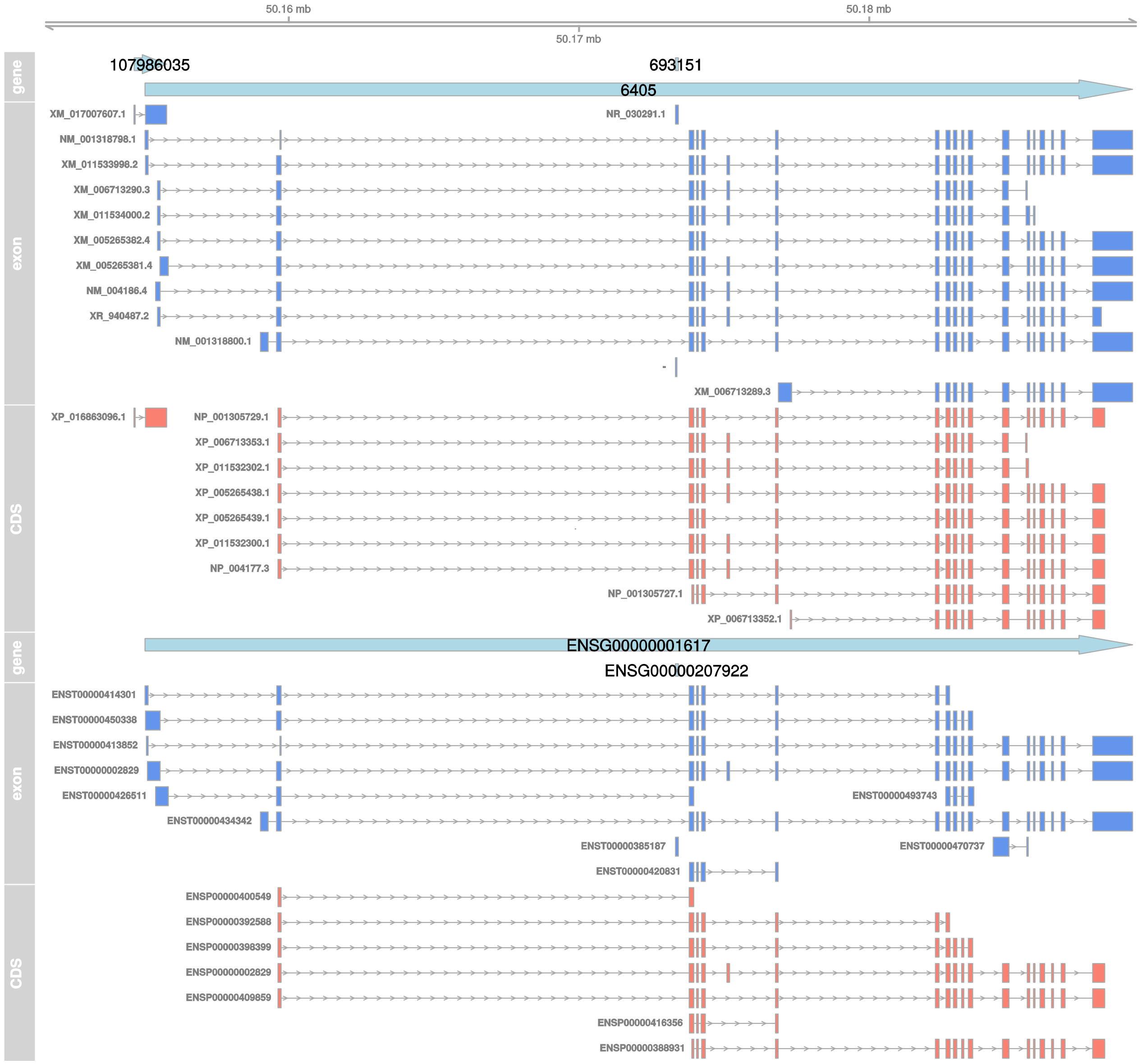

EntrezGeneID	EnsemblGeneID	GeneOL	ExonOL	CDSOL	SSOL
693151	ENSG00000207922	100.0000000	60.10638	0	100.00000
6405	ENSG00000001617	100.0000000	98.59237	100	81.48148
107986035	ENSG00000001617	2.1653200	100.00000	0	0.00000
693151	ENSG00000001617	0.2762187	0.00000	0	0.00000
6405	ENSG00000207922	0.2762187	0.00000	0	0.00000

Supplementary Figure S12. Multiple overlapping genes.

This case shows that Ensembl sometimes annotated multiple genes to identical start positions. The problem is again the correct assignment to NCBI, which was manually corrected.

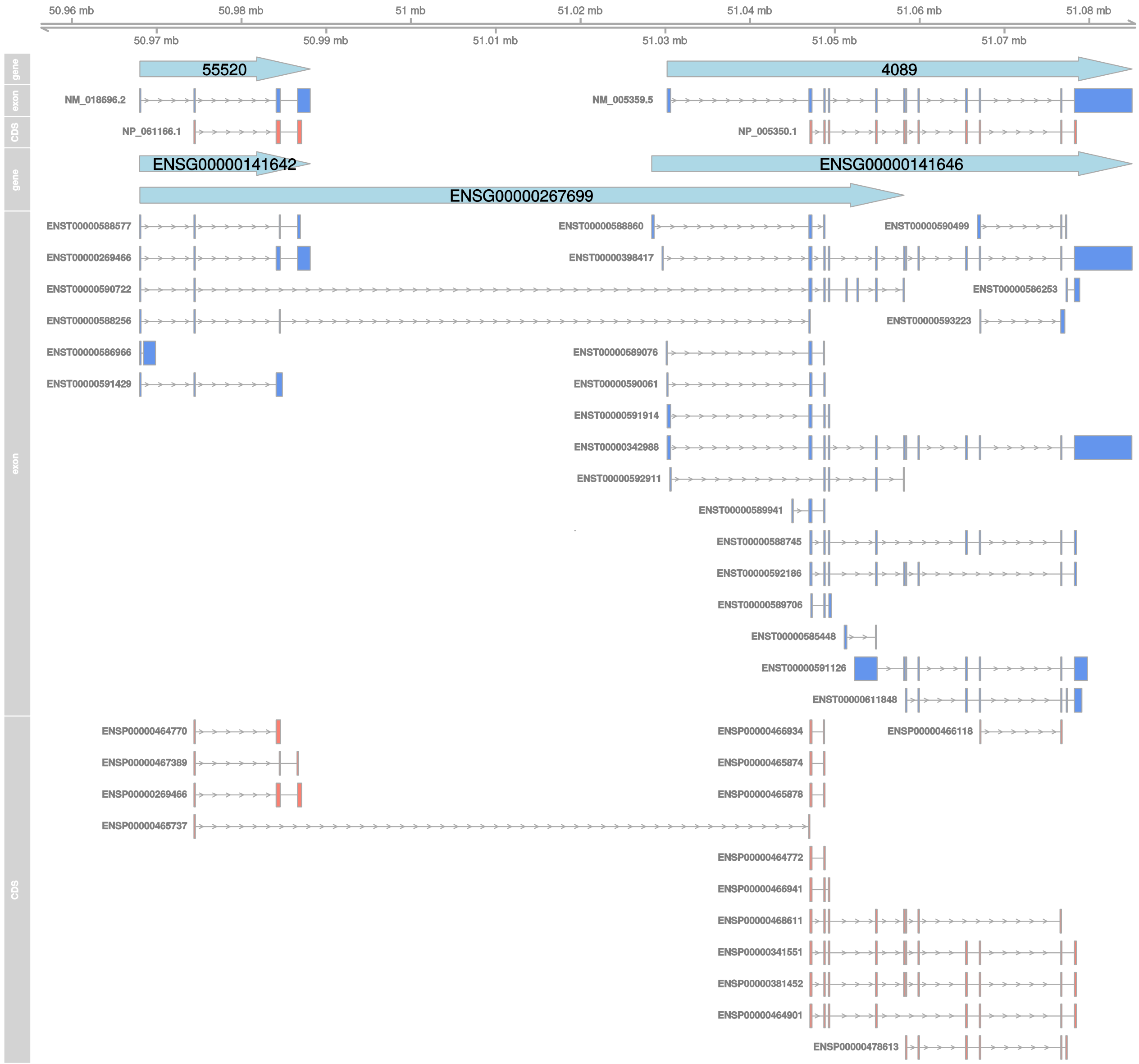

EntrezGeneID	EnsemblGeneID	GeneOL	ExonOL	CDSOL	SSOL
55520	ENSG00000141642	99.86588	100.00000	100	75.00000
4089	ENSG00000141646	96.78387	100.00000	100	95.83333
4089	ENSG00000267699	23.86861	83.33333	0	40.90909
55520	ENSG00000267699	22.30383	72.15100	100	50.00000

Supplementary Figure S13. Analysis of missing identifier pairs (IDP).

The bar plots show the number of ID pairs not identify by the AnnOverlappeR. The color code represents explanations why the ID pairs was not found or could not be found. Explanations (dup filter: removed by the duplication filter, homolog seq: Sequences were similar but not located in the identical genomic position, low OL: ID pair was removed because of a too small overlap, not in both: not found in GFF and GTF file, not in GFF, not in GTF). Compared database sources (Biomart: Ensembl BioMart, NCBI: gene2ensembl, OMABrowser: entrez2ensembl, Uniprot: entrez2uniprot & ensembl2uniprot).

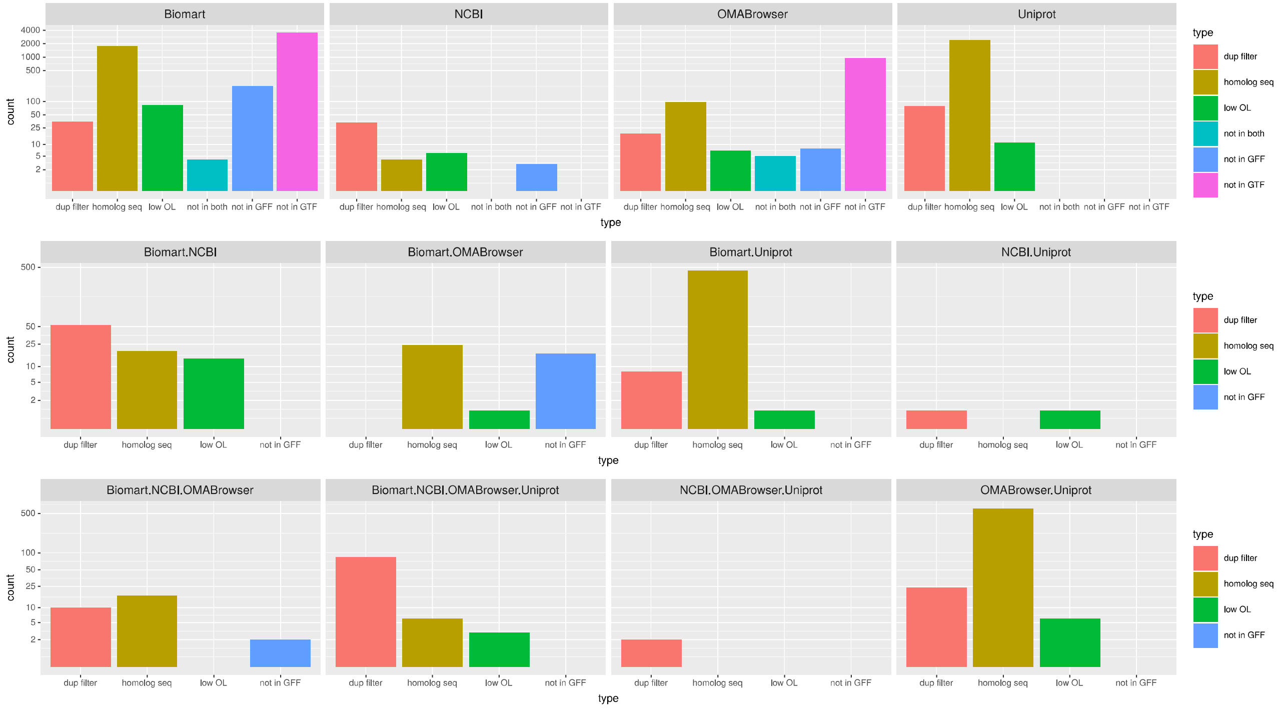

Supplementary Figure S14. Overlap of identified human orthologous genes for the test data set from the pig in comparison of 4 different database sources.

These 4 Venn diagrams show in **A** and **B** the overlapping detected human ortholog Entrez Gene IDs of 4 different database sources (Ensembl: Ensembl Compara, OMAB_AOL: OMABrowser plus AnnOverlappeR, OMAB: OMABrowser, and MAdb) separated by up- and down-regulated DEGs in porcine (pig) endometrium. Figure parts **C** and **D** represent the overlap of Ensembl gene IDs of human orthologs of three database-sources also separated into up- and down-regulated DEGs.

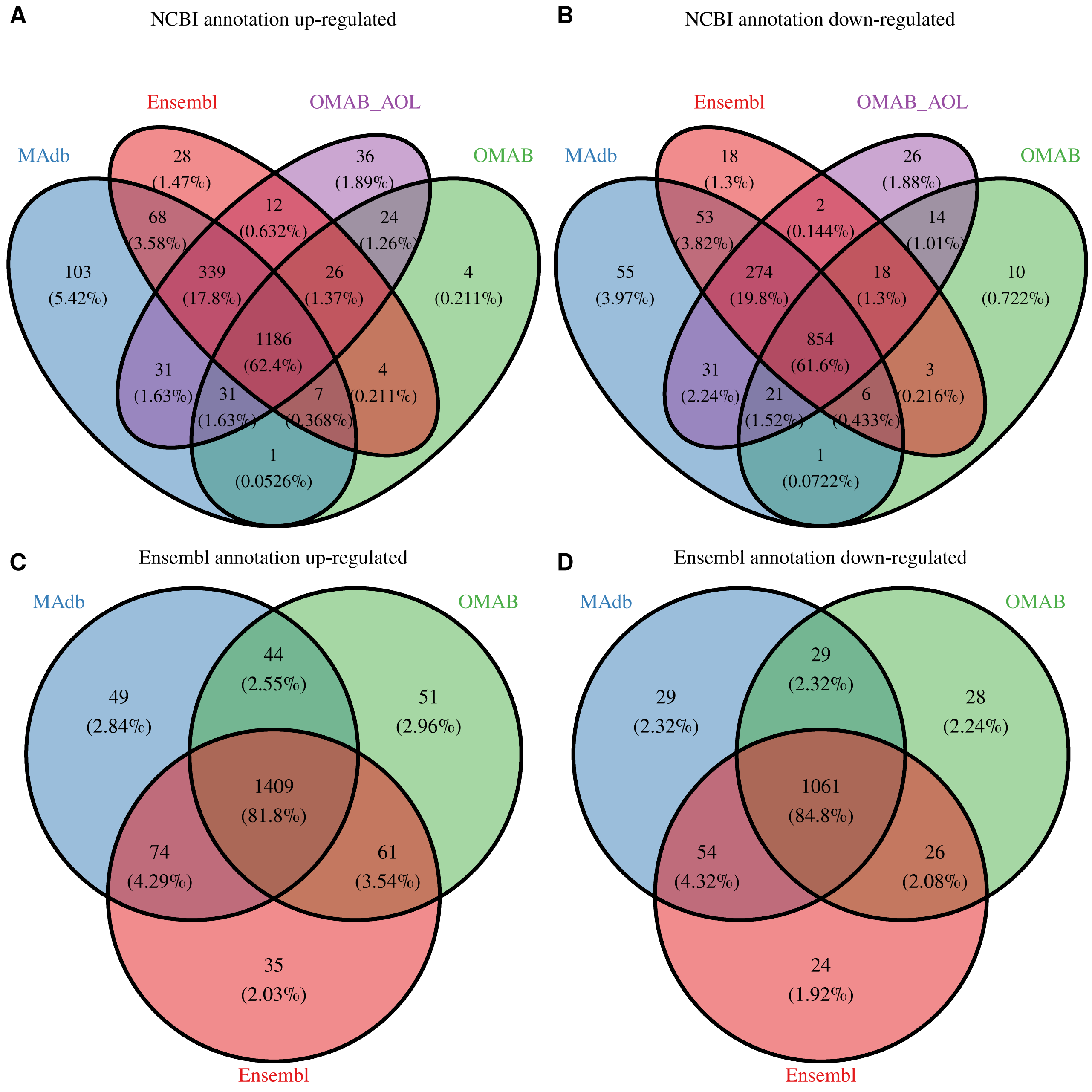

Table S1. BLASTn parameters.

**parameter**	**value**
-num_threads	6
-outfmt	7 qseqid sseqid pident length mismatch gapopen qstart qend sstart send evalue bitscore qcovs
-dust	no
-evalue	1.00E-20
-word_size	7
-max_target_seqs	1
-task	blastn
-strand	plus

Table S2. Number of gene for all seven species per type of gene.

The first table shows gene information from the NCBI (GENE_INFO), the second table shows all genes with at least one ortholog retrieved from MAdb. The third table represents the percentage of genes that have an ortholog in MAdb.

**GENE_INFO**	**protein-coding**	**ncRNA**	**pseudo**	**other**	**tRNA**	**rRNA**	**scRNA**	**snoRNA**	**snRNA**	**unknown**	**biological-region**	**sum**
bos taurus	23134	6062	5000	259	1638	21	0	634	978	20058	0	57784
canis familiaris	20422	11325	5379	170	397	0	0	0	0	8	0	37701
equus caballus	21498	7551	2858	279	474	12	0	447	432	20	0	33571
homo sapiens	20214	17276	16436	855	599	59	4	541	64	1770	3625	61443
mus musculus	27333	15412	10758	2648	523	47	13	132	20	10530	751	68167
oryctolagus cuniculus	20803	3943	5382	250	485	3	0	3	0	4	0	30873
sus scrofa	21512	6061	3090	142	510	2	0	0	0	14787	0	46104
**Ortholog hit found in MAdb**	**protein-coding**	**ncRNA**	**pseudo**	**other**	**tRNA**	**rRNA**	**scRNA**	**snoRNA**	**snRNA**	**unknown**	**biological-region**	**sum**
bos taurus	20033	621	687	89	0	5	0	473	609	50	0	22567
canis familiaris	18947	539	592	90	0	0	0	0	0	4	0	20172
equus caballus	20215	620	345	180	0	9	0	394	350	15	0	22128
homo sapiens	19099	1008	1050	333	0	15	2	374	28	7	0	21916
mus musculus	20434	651	292	463	0	16	2	123	12	36	0	22029
oryctolagus cuniculus	18807	131	718	156	0	1	0	3	0	1	0	19817
sus scrofa	19782	515	940	123	0	2	0	0	0	5	0	21367
**percent**	**protein-coding**	**ncRNA**	**pseudo**	**other**	**tRNA**	**rRNA**	**scRNA**	**snoRNA**	**snRNA**	**unknown**	**biological-region**	-
bos taurus	86.6	10.2	13.7	34.4	0.0	23.8	0.0	74.6	62.3	0.2	0.0	-
canis familiaris	92.8	4.8	11.0	52.9	0.0	0.0	0.0	0.0	0.0	50.0	0.0	-
equus caballus	94.0	8.2	12.1	64.5	0.0	75.0	0.0	88.1	81.0	75.0	0.0	-
homo sapiens	94.5	5.8	6.4	38.9	0.0	25.4	50.0	69.1	43.8	0.4	0.0	-
mus musculus	74.8	4.2	2.7	17.5	0.0	34.0	15.4	93.2	60.0	0.3	0.0	-
oryctolagus	90.4	3.3	13.3	62.4	0.0	33.3	0.0	100.0	0.0	25.0	0.0	-

cuniculus												
sus scrofa	92.0	8.5	30.4	86.6	0.0	100.0	0.0	0.0	0.0	0.0	0.0	-
average	89.3	6.4	12.8	51.0	0.0	41.7	9.3	60.7	35.3	21.6	0.0	-
